# HIV and HCV augments inflammatory responses through increased TREM-1 expression and signaling in Kupffer and Myeloid cells

**DOI:** 10.1371/journal.ppat.1007883

**Published:** 2019-07-01

**Authors:** Jinhee Hyun, Robert S. McMahon, Anna L. Lang, Jasmine S. Edwards, Alejandro Dmitar Badilla, Morgan E. Greene, Geoffrey W. Stone, Suresh Pallikkuth, Mario Stevenson, Derek M. Dykxhoorn, Shyam Kottilil, Savita Pahwa, Emmanuel Thomas

**Affiliations:** 1 Department of Microbiology and Immunology, University of Miami Miller School of Medicine, Miami, FL, United States of America; 2 Department of Medicine, University of Miami Miller School of Medicine, Miami, Florida, United States of America; 3 Dr. John T. Macdonald Foundation Department of Human Genetics, Miller School of Medicine, University of Miami, Miami FL, United States of America; 4 Institute of Human Virology, University of Maryland School of Medicine, Baltimore, Maryland, United States of America; Vaccine Research Center, UNITED STATES

## Abstract

Chronic infection with human immunodeficiency virus (HIV) and hepatitis C virus (HCV) affects an estimated 35 million and 75 million individuals worldwide, respectively. These viruses induce persistent inflammation which often drives the development or progression of organ-specific diseases and even cancer including Hepatocellular Carcinoma (HCC). In this study, we sought to examine inflammatory responses following HIV or HCV stimulation of macrophages or Kupffer cells (KCs), that may contribute to virus mediated inflammation and subsequent liver disease. KCs are liver-resident macrophages and reports have provided evidence that HIV can stimulate and infect them. In order to characterize HIV-intrinsic innate immune responses that may occur in the liver, we performed microarray analyses on KCs following HIV stimulation. Our data demonstrate that KCs upregulate several innate immune signaling pathways involved in inflammation, myeloid cell maturation, stellate cell activation, and Triggering Receptor Expressed on Myeloid cells 1 (TREM1) signaling. TREM1 is a member of the immunoglobulin superfamily of receptors and it is reported to be involved in systemic inflammatory responses due to its ability to amplify activation of host defense signaling pathways. Our data demonstrate that stimulation of KCs with HIV or HCV induces the upregulation of TREM1. Additionally, HIV viral proteins can upregulate expression of TREM1 mRNA through NF-кB signaling. Furthermore, activation of the TREM1 signaling pathway, with a targeted agonist, increased HIV or HCV-mediated inflammatory responses in macrophages due to enhanced activation of the ERK1/2 signaling cascade. Silencing TREM1 dampened inflammatory immune responses elicited by HIV or HCV stimulation. Finally, HIV and HCV infected patients exhibit higher expression and frequency of TREM1 and CD68 positive cells. Taken together, TREM1 induction by HIV contributes to chronic inflammation in the liver and targeting TREM1 signaling may be a therapeutic option to minimize HIV induced chronic inflammation.

## Author summary

Although HIV antiviral therapy has limited the progression to AIDS in infected patients, there is still significant morbidity and mortality from HIV-driven diseases due to sustained inflammation. In this study, we sought to elucidate how HIV and HCV could impact inflammation in the liver and cause progressive liver disease that can eventually lead to cirrhosis and liver cancer. We found that HIV upregulates the inflammatory response amplifier, TREM1, in primary Kupffer Cells (KCs) that are liver-resident macrophages. Enhanced TREM1 expression subsequently is involved in augmented immune responses triggered by HIV or HCV. Additionally, our data demonstrates that blocking TREM1 expression reduces inflammatory responses mediated by HIV or HCV stimulation. Ultimately, our understanding of this mechanism may yield additional therapeutic strategies to help infected patients and give insight into inflammation driven liver cancer.

## Introduction

Cell-intrinsic innate immune responses provide the first line of defense against invading viral pathogens. These early innate immune responses not only blunt the initial spread of infection but also activate the adaptive immune system and secondary host defense mechanisms. However, the immune reaction must be controlled and balanced in order to maintain immune homeostasis and to prevent subsequent tissue injury arising from chronic inflammation [1–3]. Human immunodeficiency virus (HIV) and hepatitis C virus (HCV) are RNA viruses capable of inducing and sustaining systemic inflammation [4,5]. HCV infects hepatocytes and subsequently stimulates robust hepatic antiviral immune responses by stimulating the secretion of type III interferons, cytokines, and chemokines [6–8]. During HCV infection, cytokines and chemokines drive hepatic inflammation by recruiting mononuclear immune cells, including T cells and monocytes, into the liver. These inflammatory responses are thought to cause hepatocyte damage through several mechanisms that ultimately lead to liver fibrosis, cirrhosis and hepatocellular carcinoma (HCC) [9,10]. Distinct from HCV, HIV establishes infection within CD4 expressing immune cells such as CD4+ T lymphocytes and macrophages [5,11]. Infection with HIV causes significant perturbations within the immune system including CD4 T cell depletion and the induction of systemic inflammation through bacterial translocation across the gut mucosa [12–14]. Since the increased level of microbial products and metabolites can penetrate the liver, HIV infection may indirectly impact hepatic parenchymal and nonparenchymal cells. It has recently been reported that microbial pathogen-associated molecular patterns (PAMPs) from the leaky gut are sensed by hepatocytes, hepatic stellate cells (HSCs) and Kupffer cells (KCs) to trigger proinflammatory and profibrotic signaling [15]. Several studies have also demonstrated that HIV directly infects nonparenchymal liver cells, including HSCs and KCs, which constitute 4–8% of the total liver cell population [16]. Infection of KCs with HIV has been demonstrated by in vitro viral replication experiments and by detection of viremia in HIV infected patients or SIV infected macaques where KCs where specifically analyzed [17–20]. Subsequent interaction with HIV not only results in stimulating proinflammatory responses but also in altering KC phenotype and function [20,21]. In this and related inflammatory processes, pathogen recognition receptors (PRRs) including Toll like receptors (TLRs), Retinoic acid-inducible gene-I (RIG-I)-like receptors (RLRs), and Nucleotide oligomerization domain (NOD)-like receptors (NLRs) all have been shown to play an essential role in hepatic innate immune responses triggered by HIV PAMPs [22]. These receptors are expressed at a low level to maintain the tolerogenic state within the liver. However, upregulation of PRRs has been reported to increase hepatic inflammation and is linked to the progression of chronic liver disease [23,24]. This suggests that augmented hepatic innate immune responses may play a critical role in liver pathology in patients with HIV and HCV infection.

Similar to pathogen recognition receptors (PRRs), TREM1 is involved in innate immune and inflammatory responses. TREM1 is a 30KDa V-type IgG orphan immunoreceptor extensively expressed on the surface of neutrophils, monocytes, and macrophages. Activation of TREM1 signaling by crosslinking of the ligand results in production of tumor necrosis factor alpha (TNFα), IL-18 and C-C Motif Chemokine Ligand 2 (CCL2) through the adaptor DNAX-Activation Protein 12 (DAP12) [25,26]. This activation amplifies proinflammatory responses induced by TLR or NLR ligands [27]. Accordingly, TREM1 expression and activation has been reported to be linked to pathological conditions including rheumatoid arthritis, inflammatory bowel disease, and liver diseases including hepatocellular carcinoma [28–30].

Therefore, the current study sought to investigate the mechanisms through which HIV or HCV infection impacts innate immune responses in KCs and macrophages that express several distinct PRRs and orchestrate innate and adaptive immune responses. Our data demonstrate that stimulation with HIV or HCV induces robust inflammatory immune responses in KCs and monocyte derived macrophages (MDMs) independent of viral infection. We also show augmented inflammatory responses through TREM1 upregulation by HIV or HCV exposure. Furthermore, we confirm that increased TREM1 signaling stimulates inflammatory cytokine production from macrophages. These findings demonstrate that TREM1 contributes to inflammatory responses observed in KCs and macrophages during viral infection and that suppression of TREM1 signaling may be used as strategy to attenuate virus-induced liver disease progression and the subsequent development of HCC.

## Results

### Kupffer cells (KCs) express PRRs and innate antiviral signaling molecules that activate immune responses upon stimulation with viral mimetics

**Fig 1:**
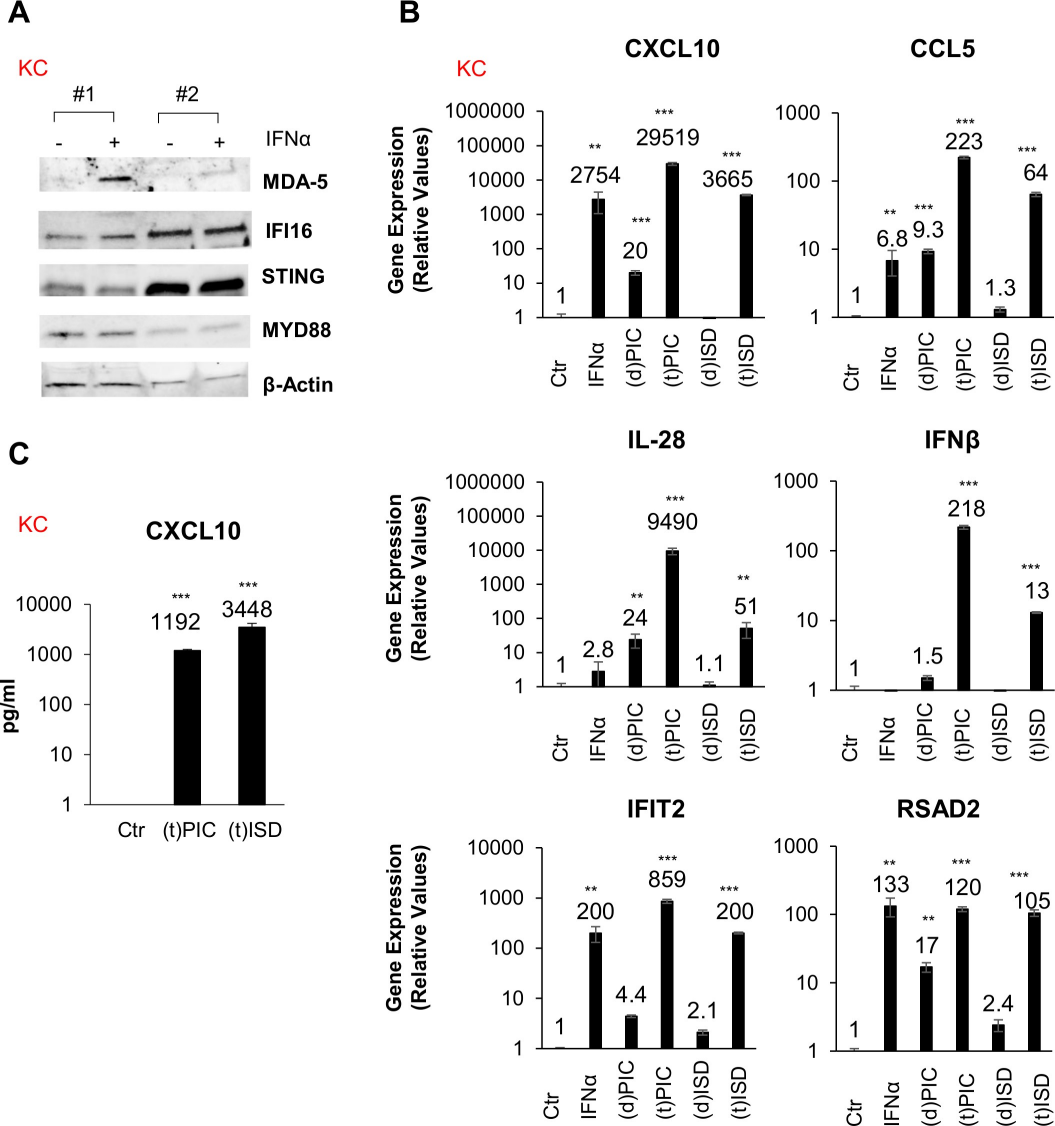
Primary human KCs induce robust innate immune responses following stimulation with viral mimetics. (A) Protein levels of MDA-5, IFI16, STING, and MYD88 were examined via Western blot in Kupffer cells (KCs, n = 2) ± IFNα (1000 U/ml). *β*-actin was used as internal control. (B) KCs were stimulated directly (d) or transfected (t) with viral mimetics poly (I:C) (PIC, 6 μg/ml) and ISD (6 μg/ml). Gene expression for innate antiviral responses and chemokines CXCL10, CCL5, IFNβ, IL-28, IFIT2, and RSAD2 are shown from qPCR analysis. (C) CXCL10 protein expression measured by ELISA in poly (I:C) or ISD transfected (t) KC. All treatments were performed for 24 hours. For qPCR, results are shown as fold induction compared to control samples after normalizing with 18S internal control. Data from repeated experiments were averaged and are expressed as means ± SD. *P≤0.05, **P≤0.01, ***P≤0.001; ns, non-significant.

To characterize intrinsic innate immune responses in primary KCs, we first compared the basal expression of genes that are essential for intrinsic innate immunity in macrophages. We used THP1 monocytes for comparison as this cell line is representative of macrophage immune responses. Importantly, human primary cells were also used for comparison and included MDMs and non-parenchymal liver cells (NPCs). Quantitative PCR (qPCR) analysis revealed that KCs, NPCs and MDMs express higher endogenous mRNA levels of (RIG-I), melanoma differentiation-associated gene 5 (MDA-5), Interferon Gamma Inducible Protein 16 (IFI16), TLR3, and Myeloid Differentiation Primary Response 88 (MYD88) when compared to THP1 monocytes and hepatocytes (primary human hepatocytes and HepaRG). Conversely, the levels of cyclic GMP-AMP synthase (cGAS) and Stimulator of interferon genes (STING) were higher in the THP1 monocytes, but the expression of these cytosolic DNA sensors was lower in hepatocytes. (S1A–S1C Fig). We next examined the basal protein expression levels of several PRRs and their adaptor proteins. Since some genes were undetectable at baseline, we used THP1, MDMs, NPCs, and KCs that were first stimulated with interferon (IFN)α. Treatment with IFNα increased protein expression of RIG-I, MDA-5, IFI16, and MYD88 in NPCs and MDMs. In KCs, we observed the upregulation of MDA-5 following stimulation with IFNα. However, STING and MAVS levels did not increase in THP1, MDMs, NPCs, and KCs following stimulation with IFNα (Fig 1A and S1D Fig).

To further investigate innate immune responses in KCs, the cells were transfected (t) with poly(I:C) or interferon stimulatory DNA (ISD) for 24 hours. Then, qPCR analysis was performed in order to examine mRNA expression of genes involved innate immune responses, including C-X-C Motif Chemokine Ligand 10 (CXCL10), C-C Motif Chemokine Ligand 5 (CCL5), IFNβ, IL-28, Interferon Induced Protein With Tetratricopeptide Repeats 2 (IFIT2), and radical S-adenosyl methionine domain-containing protein 2 (RSAD2). Transfection of viral mimetics significantly increased innate immune signaling mRNA expression, whereas direct addition (d) of poly(I:C) or ISD to the culture media elicited marginal increases in mRNA expression (Fig 1B). Next, to identify secreted chemokines from KCs, the supernatants from cells transfected with poly(I:C) or ISD were analyzed via enzyme-linked immunosorbent assay (ELISA) analyses. Interestingly, stimulation with both RNA and DNA viral mimetics induced secretion of the chemokine CXCL10 from KCs (Fig 1C). Collectively, these data indicate that KCs possess functional innate immune signaling pathways, as do other macrophage cell types, and that they are capable of mounting antiviral immune responses following stimulation with diverse viral mimetics.

### Primary KCs demonstrate activation of inflammatory responses when stimulated with HIV

Although HIV has been shown to directly infect KCs *in vitro* and viral HIV RNA has been detected in the intracellular compartment of KCs isolated from HIV infected patients, the innate immune response to HIV remains to be fully clarified [17,18,21,31]. To evaluate whether HIV stimulates inflammatory responses in KCs, in an unbiased analysis, we exposed KCs to HIV-IIIB [multiplicity of infection (MOI) = 2] for 24 hours and performed microarray analyses. When comparing HIV-stimulated KCs to untreated controls, we identified HIV regulated genes utilizing a cutoff of P<0.05 and a fold-change greater than 2.0 with a false discovery rate (FDR) cutoff of 0.05.

**Fig 2:**
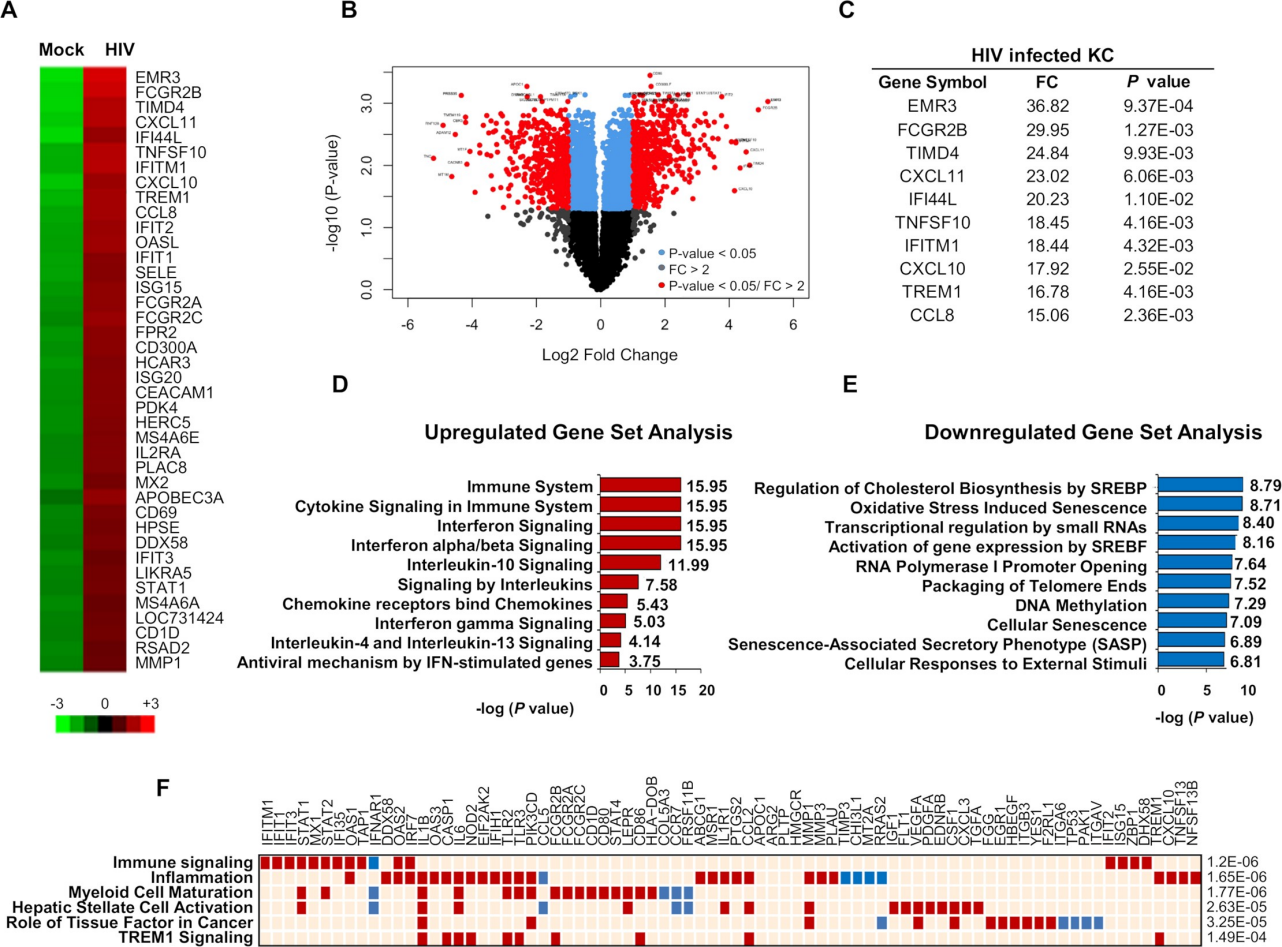
KC exposure to HIV induces upregulation of proinflammatory genes. (A) Top 40 upregulated genes in KC exposed to HIV-IIIB for 24 hours at an MOI of 2. (B) Volcano plot showing changes in gene expression stratified by log fold change and p-value (C) Top 10 genes with fold change (FC) greater than 15 (*P≤0.05). (D) Pathway Analysis showing the top 10 upregulated signaling pathways. (E) Pathway analysis showing the top 10 downregulated signaling pathways. (F) Checkerboard plot shows the top 6 enriched Ingenuity pathways. Data are from one experiment with technical replicates.

Our microarray results demonstrate that HIV stimulation altered gene expression of various inflammatory and antiviral genes at the transcriptional level (Fig 2A and 2B). As shown in Fig 2C, upregulation of chemokines and interferon-stimulated genes were observed with the top 10 genes being induced greater than 15 fold. In addition, pathway analysis was performed using the Reactome Pathway Knowledgebase and verified using Qaigen’s Ingenuity Pathway Analysis software. Our data demonstrated that several general innate immune response pathways were upregulated in HIV-treated KCs, including immune signaling, inflammation, myeloid maturation, stellate cell activation and TREM1 signaling (Fig 2D, 2E and 2F). Overall, our microarray data suggest that HIV induces an inflammatory gene signature in KCs that may contribute to liver disease progression.

### Production of proinflammatory cytokines and chemokines from KCs and monocyte derived macrophages when stimulated with HIV

**Fig 3:**
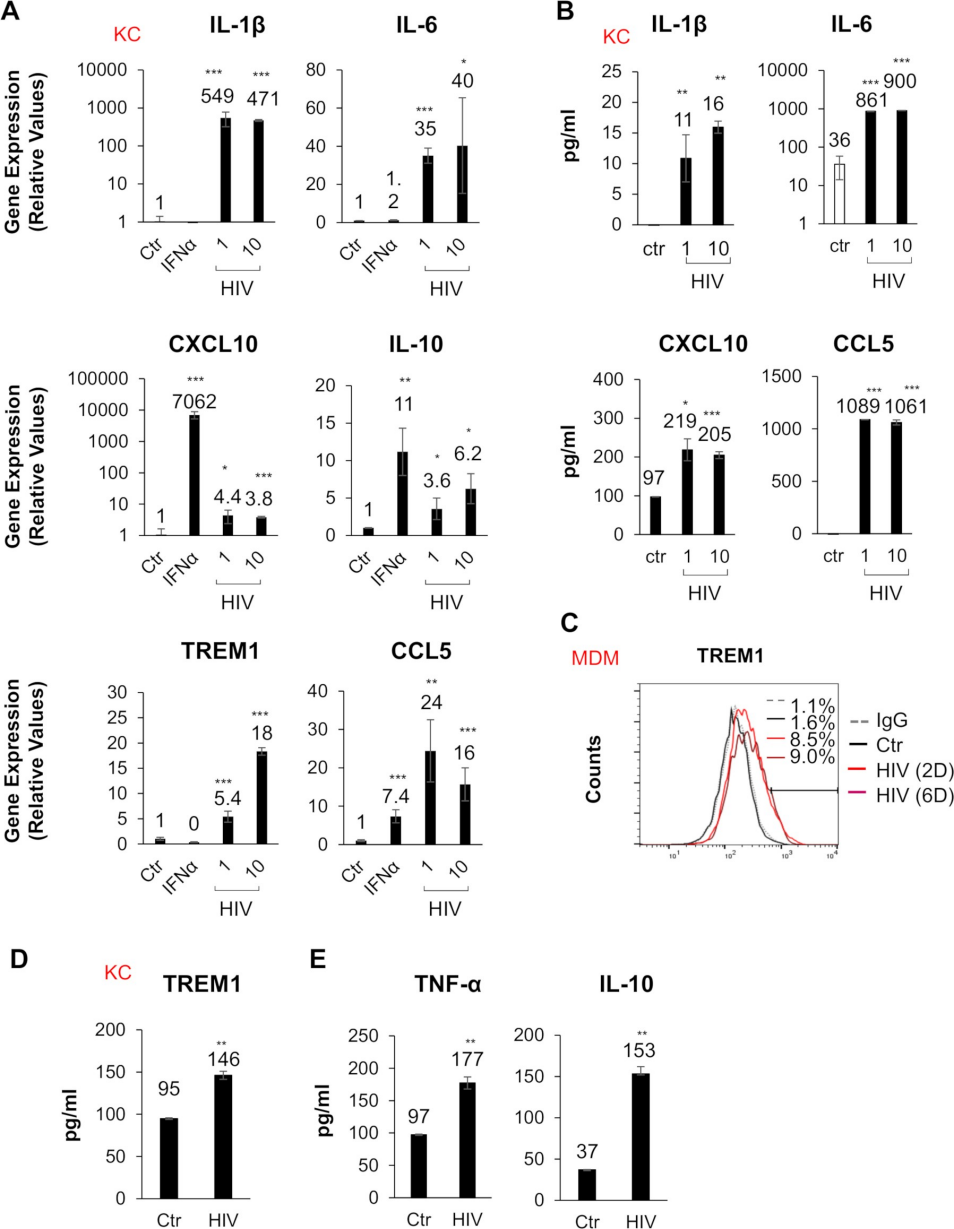
HIV increases production of proinflammatory cytokines and chemokines in KCs. (A) KCs were stimulated with increasing MOI (1, 10) of HIV or with IFNα (10 U/mL). qPCR analysis of gene expression for IL-1β, IL-6, TREM1, CCL5, CXCL10, and IL-10 are shown. (B) Supernatants from HIV-stimulated KCs were collected and analyzed by ELISA for levels of IL-1β, IL-6, CXCL10, and CCL5. (C) MDMs were exposed to HIV (MOI = 1) for either 2 or 6 days. Flow cytometry analysis of TREM1 surface expression was analyzed. (D) KCs were stimulated with HIV for 48 hours and cell lysate were subject to ELISA for TREM1. (E) Supernatants from HIV-stimulated KCs (MOI = 1 for 48 hours) were analyzed by multiplex cytokine ELISA array. Data for TNFα and IL-10 are shown compared to control samples. For qPCR, results are shown as fold induction compared to control samples after normalizing with 18S internal control. Data from repeated experiments were averaged and are expressed as means ± SD. *P≤0.05, **P≤0.01, ***P≤0.001; ns, non-significant.

To validate our microarray data, we treated KCs with increasing MOI of HIV, for 24 hours, and assessed changes in expression of inflammatory genes by qPCR analysis. Several proinflammatory cytokines and chemokines including IL-1β, IL-6, CXCL10 and CCL5 were upregulated consistent with the microarray data (Fig 3A). We also validated protein expression levels of these genes by ELISA with supernatants obtained from HIV treated KCs (Fig 3B). Importantly, TREM1 protein upregulation was confirmed (Fig 3C and 3D) in MDMs and KCs following stimulation with HIV by flow cytometry or ELISA analysis. Additionally, we observed the upregulation of several interferon stimulated genes (S2A Fig). Finally, we confirmed that the viral particle was critical for stimulation since media obtained directly from the isolated viral stock, by filtration, did not induce CXCL10 or TREM1 gene expression (S2B Fig). To further confirm upregulation of this inflammatory gene signature in other macrophage/monocyte cell types, we stimulated human MDMs with HIV. qPCR analysis demonstrated that HIV stimulation in MDMs elicited the upregulation of similar inflammatory cytokines and chemokines (S3A Fig) as observed in the stimulated KCs. We also verified the purity of the macrophages and addressed the possibility of contaminating dendritic cells and neutrophils in our MDM preparations by quantitating the levels of CD68, CD15, and CD209 expression (S2C Fig). Next, we examined the expression of other key inflammatory proteins through a cytokine multiplex ELISA array. The levels of sixteen protein targets were measured in the supernatants from HIV treated KCs. These results demonstrated that HIV stimulation promotes the secretion of cytokines, such as TNF-α and IL-10 in KCs compared to untreated controls (Fig 3E). Overall, these data suggest that HIV simulation may drive inflammatory responses through the stimulation of liver and other tissue resident macrophages.

### KCs and monocyte derived macrophages also secrete proinflammatory cytokines and chemokines in response to HCV

Next, we evaluated inflammatory responses to HCV in KCs and MDMs. Although KCs do not support HCV replication, several studies have reported that HCV stimulation induces proinflammatory cytokine and chemokine secretion, including IL-1β [7,32,33]. We subsequently assessed changes in gene expression by qPCR analysis utilizing KCs or MDMs stimulated with HCV. Similar to HIV, HCV increased the transcription of IL-1β, IL-6, and CCL5 (S4A Fig). We also detected upregulation of the anti-inflammatory cytokine IL-10. In addition, we confirmed upregulation of these genes following HCV stimulation in MDMs (S5A Fig). Next, we analyzed the production of proinflammatory cytokines, including IL-1β, IL-6 and the chemokine CCL5, through ELISA on supernatants from HCV stimulated KCs (S4B Fig). Cell surface expression of TREM1 was also quantitated on MDMs following stimulation with HCV using flow cytometry (S4C Fig). Together, our results demonstrate that HCV also induces a proinflammatory response in hepatic and non-hepatic macrophages.

### Inflammatory responses in macrophages stimulated with HIV and HCV are infection independent

Since we observed broad inflammatory responses in virus stimulated macrophages, the kinetics of gene induction following HIV or HCV stimulation was investigated. Several genes were upregulated in KCs following 3 hours of viral exposure. mRNA expression of inflammatory cytokines such as IL-1β and IL6 were elevated at 24 hours post stimulation; however, CCL5 peaked at 8 hours and decreased 24 hours after the addition of virus. In contrast, TREM1 gradually increased its expression and peaked at 24 hours after HIV or HCV stimulation (S3B and S5B Figs). Next, to determine if the inflammatory signature was dependent on viral infection, anti-CD4 or anti-CD81 receptor targeted neutralizing antibodies were applied to MDMs prior to HIV or HCV exposure and the treated cells were then tested for gene upregulation. Regardless of treatment with a non-specific control IgG or the specific receptor IgG antagonist, viral stimulation was able to upregulate inflammatory genes (S3C and S5C Figs). This suggests that viral infection and replication was not necessary to trigger the inflammatory response. To further determine whether viral replication was required for the observed inflammatory responses, we stimulated MDMs with UV inactivated virus (S3F and S5E Figs). We also examined virus endocytosis using p24 immunostaining and observed that UV irradiated viral particles were engulfed by macrophages (S3E Fig). Our qPCR analysis revealed that there were no differences in inflammatory gene induction between UV treated or untreated HIV or HCV (S3D and S5D Figs). These data suggest that macrophage responses to HIV or HCV are independent of replication during the early stages of viral exposure.

### TREM1 gene induction occurs by HIV and HCV through endocytosis

**Fig 4:**
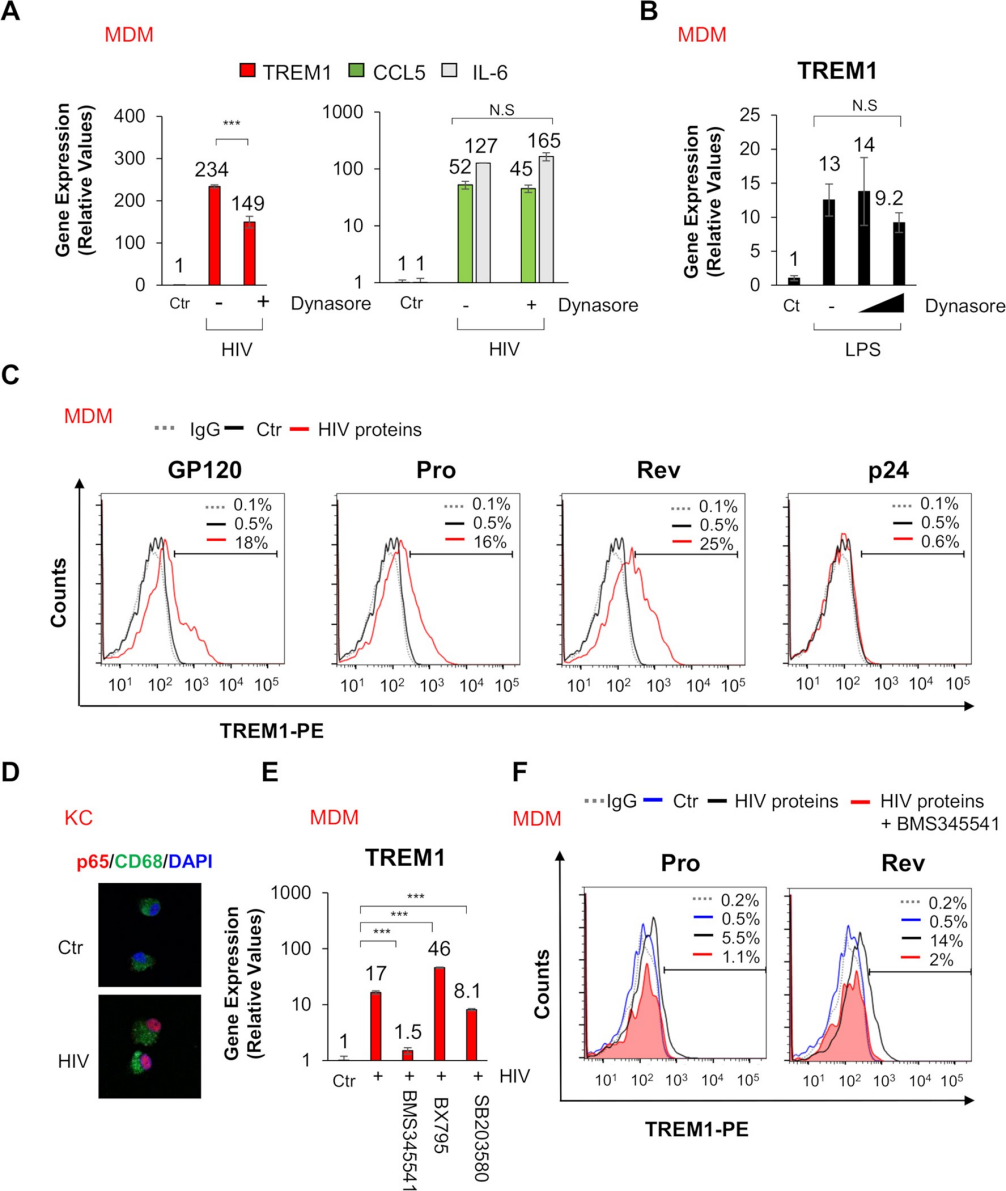
HIV viral proteins upregulate TREM1 gene expression during HIV stimulation. (A) MDMs were stimulated with HIV ± Dynasore (endocytosis inhibitor, 80 μM). Gene expression of TREM1, CCL5, and IL-6 was analyzed by qPCR. (B) MDMs were stimulated with LPS (10ug/ml) ± Dynasore (80, 160uM) and gene expression of TREM1 is shown. (C) MDMs were stimulated with HIV viral proteins gp120, Pro, Rev, and p24 (10 μg for 24 hours). Flow cytometry analysis of TREM1 surface expression is shown compared to control or IgG treated samples. (D) Nuclear localization of p65 in KC after treating with HIV for 1 hour. (E) MDMs were stimulated with HIV ± cell signaling inhibitors BMS345541 (IKK inhibitor, 2 μM), BX795 (TBK1 inhibitor, 5 μM), SB203580 (MAPK inhibitor, 20 μM)] and qPCR analysis of TREM1 gene expression is shown. (F) Flow cytometry analysis of TREM1 surface expression in MDMs after stimulating with HIV proteins Pro and Rev ± BMS345541 is shown. HIV stimulation was performed at a MOI = 1 for 24 hours. For qPCR, results are shown as fold induction compared to control samples after normalizing with 18S internal control. Data from repeated experiments were averaged and are expressed as means ± SD. *P≤0.05, **P≤0.01, ***P≤0.001; ns, non-significant.

Recently, several studies have demonstrated that endocytosis is involved in HIV triggered inflammatory responses [33–35]. To investigate the influence of the endocytosis of virions that may subsequently trigger changes in genes expression, we utilized the small molecule Dynasore that inhibits caveolar- and clathrin-mediated pathways. The induction of several genes were unaffected, in MDMs, following treatment with Dynasore and HIV (Fig 4A). However, the induction of TREM1 gene expression was significantly decreased with Dynasore administration. Involvement of the endocytic process, in TREM1 upregulation, was specific to HIV and HCV stimulation since Dynasore administration had no effect on LPS-mediated TREM1 upregulation (Fig 4B and S6A Fig). We also tested the possibility that autocrine cytokine signaling may result in the upregulation of TREM1 in MDMs. MDMs were treated with supernatant filtrates of HIV stimulated MDMs at different time points. Importantly, the filtration step removed residual virions from the media while secretary cytokines would remain. Our results revealed that the media filtrate from HIV or HCV stimulated MDMs did not upregulate TREM1 (S6B Fig). Moreover, incubation with exogenous cytokines including TNF-α, IL-6 and IFNα did not upregulate TREM1 expression (S6C Fig).

To evaluate which viral components activate signaling that leads to TREM1 upregulation, we transfected MDMs with viral mimetics including poly(I:C), ISD, or 2’3’cGAMP. We also applied HIV genomic RNA directly to MDMs. Neither HIV genomic RNA, nor these viral mimetics stimulated the upregulation of TREM1 (S6D and S6E Fig). Since, HIV genomic RNA or these viral mimetics had no impact on TREM1 upregulation, we next determined whether HIV viral proteins could induce TREM1 gene expression. It has been reported that the transmembrane HIV envelope protein gp41 stimulates TREM1 upregulation in peripheral blood mononuclear cells (PBMCs) [36]. In addition, incubation with the recombinant HIV envelope protein gp160 and accessory protein, Tat, was shown to upregulate TREM1 in RAW 264.7 cells [37]. To determine if HIV viral proteins are capable of upregulating TREM1 gene expression in human macrophages, we stimulated MDMs with several HIV recombinant viral proteins and measured TREM1 protein expression on the cell surface by flow cytometry. In accordance with other studies, we confirmed upregulation of TREM1 by the HIV envelope protein gp120. Moreover, we observed that accessory proteins including Pro and Rev can enhance the surface expression of TREM1 (Fig 4C). However, HIV core protein p24 demonstrated no effect on cell surface expression of TREM1.

To determine which signaling pathways are responsible for TREM1 gene induction in human macrophages during HIV or HCV stimulation, we used pathway-specific inhibitors. TREM1 gene expression is regulated by several transcription factors, including nuclear factor-kappa B (NF-κB), Activator protein 1 (AP-1), and cAMP response element binding protein (CREB). Accordingly, we first confirmed that HIV stimulation induces NF-κB, activation in KCs by staining for p65 nuclear localization and then utilized the corresponding antagonists of these signaling pathways (Fig 4D). Interestingly, TREM1 gene induction was significantly decreased by the IKK complex selective inhibitor BMS345541 (Fig 4E and S6F Fig). Additionally, treatment with the MAP kinase inhibitor SB203580 partially suppressed TREM1 gene induction, suggesting that activation of AP-1 is required. However, treatment with a TANK-binding kinase 1 (TBK1) inhibitor BX795 was unable to abolish upregulation of TREM1 demonstrating the involvement of signaling pathways that are distinct from those involved in IFN production. We confirmed that cell viability was unaffected following treatment with these small molecules using the alamarBlue assay (S6G Fig). Next, we examined expression of the TREM1 protein, by flow cytometry, after treating with the HIV viral proteins Pro and Rev in the presence of the NF-KB inhibitor. Fig 4F demonstrates that TREM1 upregulation by these viral proteins was significantly decreased in cells that were treated with BMS345541. Taken together, these findings demonstrate that TREM1 gene expression is modulated by the endocytosis of virions and that HIV viral proteins induce TREM1 through NF-κB signaling.

### Activation of TREM1 signaling induces proinflammatory immune responses by HIV and HCV

**Fig 5:**
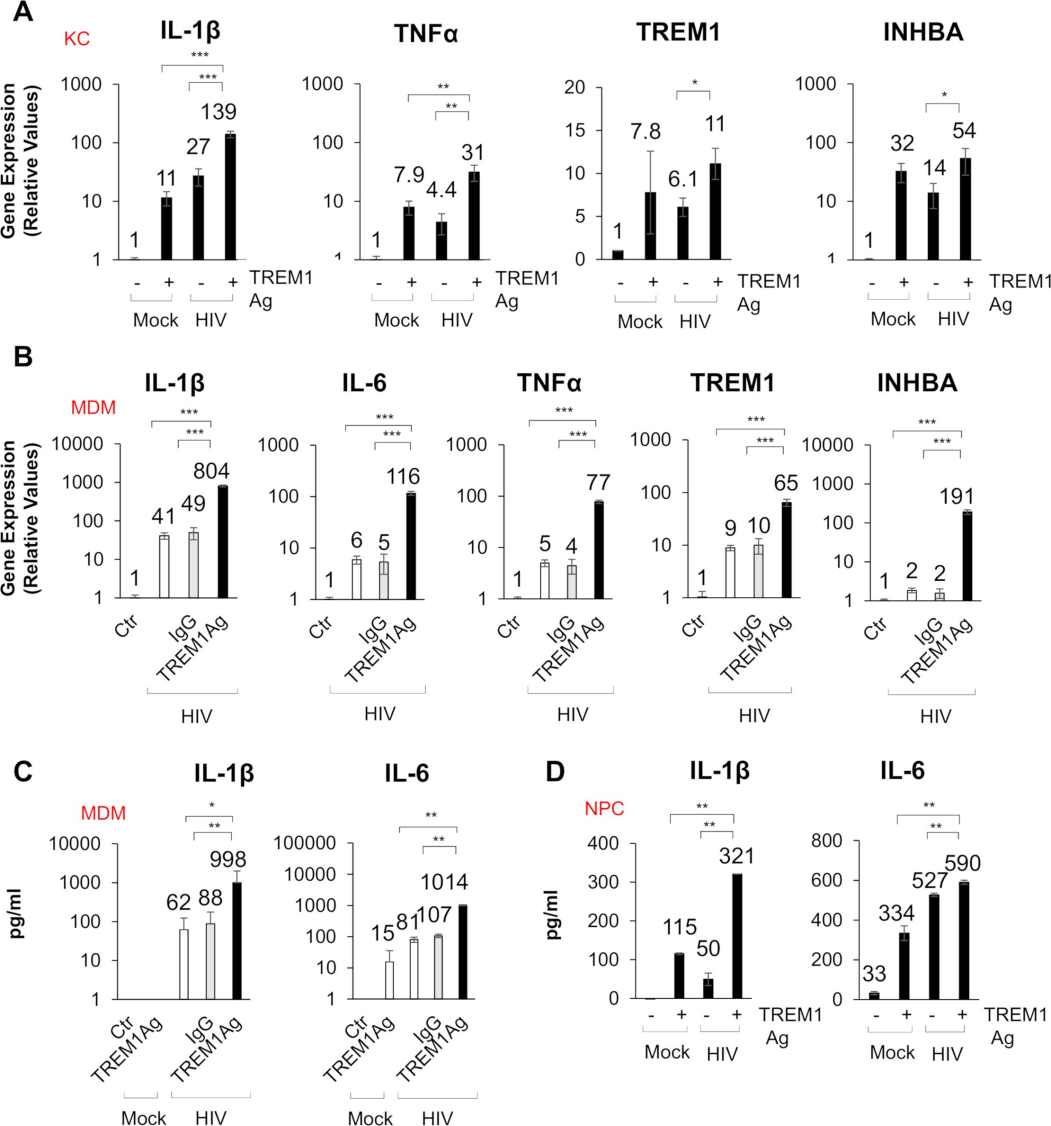
TREM1 activation enhances proinflammatory cytokine secretion from macrophages stimulated with HIV. (A) KCs were stimulated with HIV (MOI = 1, 24 hours) ± IgG (1 μg/mL) or TREM1 agonist (TREM1Ag, 1 μg/mL) and gene expression of IL-1β, TNFα, INHBA, and TREM1 are shown from qPCR analysis. (B) qPCR analysis of IL-1β, IL-6, TNFα, INHBA, and TREM1 gene expression in MDMs after stimulation with HIV (MOI = 1, 24 hours) ± IgG (1 μg/mL) or TREM1 agonist (TREM1Ag, 1 μg/mL). (C) IL-1β and IL-6 levels were analyzed by ELISA from MDMs treated in (B) and results are shown in pg/mL. (D) Protein expression of IL-1β and IL-6 measured by ELISA in NPCs with HIV in the presence or absence of TREM1 agonist. For qPCR, results are shown as fold induction compared to control samples after normalizing with 18S internal control. Data from repeated experiments were averaged and are expressed as means ± SD. *P≤0.05, **P≤0.01, ***P≤0.001; ns, non-significant.

Given that TREM1 plays a significant role in inflammatory responses, we generated a stable cell line overexpressing TREM1 using lentiviral transduction of THP1 cells to evaluate the function of TREM1 signaling during viral exposure. We confirmed TREM1 mRNA expression by qPCR analysis and cell surface expression by flow cytometry (S7A Fig). To determine if exogenous expression of TREM1 had an effect on baseline expression of genes involved in inflammatory signaling, the endogenous mRNA expression of NFKB1A, IKBKB, MYD88 and Toll Like Receptor Adaptor Molecule (TRIF) were compared. qPCR analysis demonstrated that the exogenous expression of TREM1 did not alter the gene expression of NF-κB or TLR4 signaling adaptors (S7B Fig). It has been reported that TREM1 amplifies innate immune responses through receptor mediated activation by binding its cognate ligand. Accordingly, we used an antibody agonist to stimulate TREM1 signaling that induces known target genes including Inhibin Beta A (INHBA). We treated the parental THP1 and our TREM1 overexpressing THP1 cells with the agonist to verify specificity. Following treatment with the TREM1 agonist, TREM1 expressing THP1 cells robustly upregulated expression of INHBA, while the agonist minimally affected INHBA gene induction in the parental THP1 cells (S7C Fig). Next, we investigated the role of TREM1 signaling in HIV and HCV induced immune responses. We incubated these viruses with control IgG or the TREM1 agonist in KCs or MDMs. qPCR analysis demonstrated that stimulation with HIV or HCV in combination with the TREM1 agonist enhanced the upregulation of IL-1β, IL-6, TNFα, and TREM1 itself (Fig 5A and 5B and S7D Fig). Consistent with our qPCR results, ELISA analysis demonstrated that the TREM1 agonist alone increased IL-6 production. When compared to either virus or TREM1 agonist alone, incubation with both HIV or HCV and the TREM1 agonist resulted in 10-fold higher secretion of proinflammatory cytokines (Fig 5C and 5D and S7E and S7F Fig). However, this effect was limited to inflammatory cytokines. Collectively, our data demonstrate that activation of TREM1 results in significant upregulation of IL-1β, TNFα, and IL-6 and that activation of TREM1 signaling augments inflammatory cytokine production in macrophages.

### Activated TREM1 signals through the ERK pathway in macrophages

**Fig 6:**
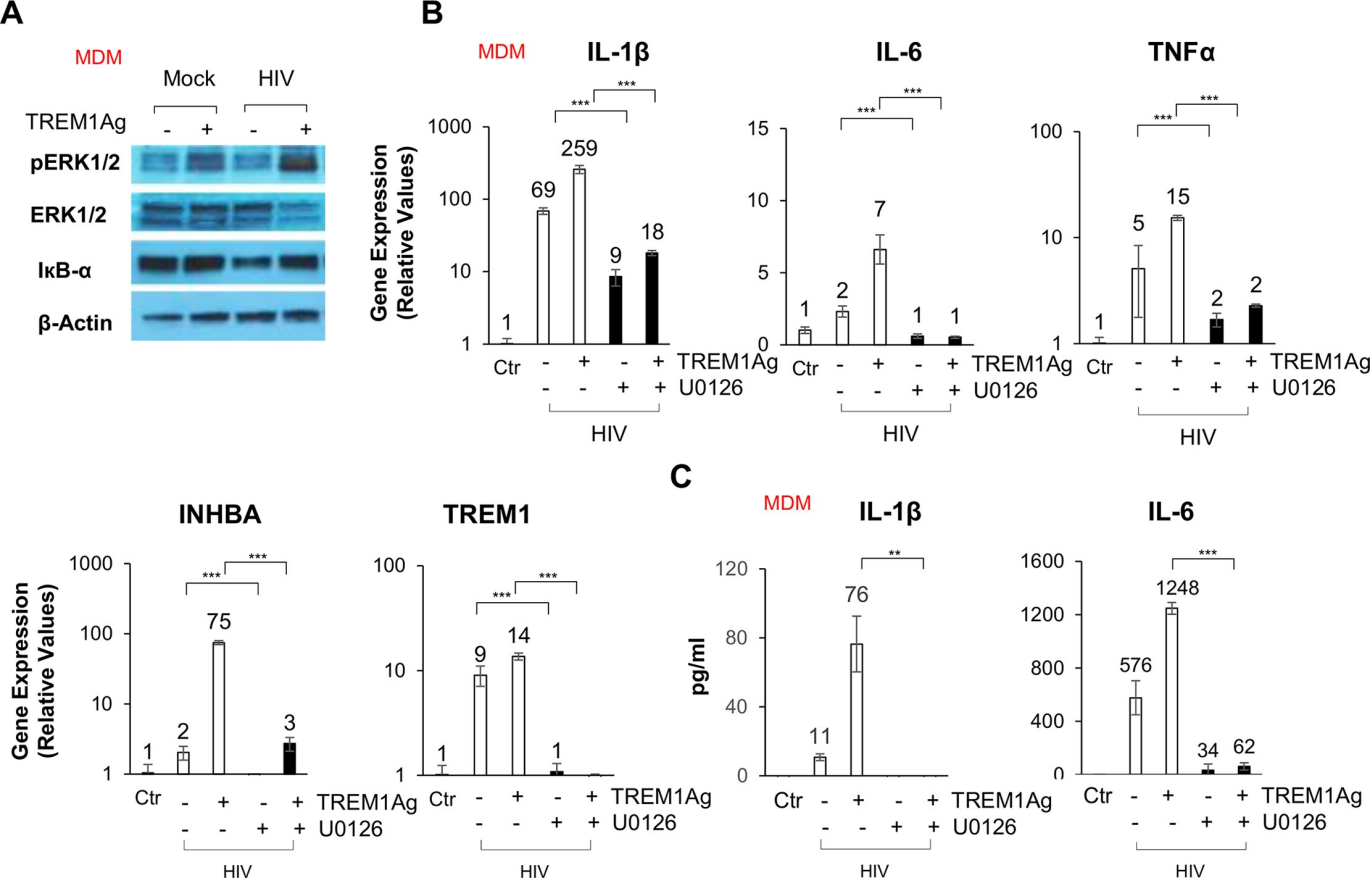
The ERK signaling pathway is essential for TREM1 mediated inflammatory responses during HIV exposure. (A) MDMs were stimulated with HIV ± TREMAg1 (1 μg/mL) and protein levels of phospho-ERK1/2, total ERK1/2, and IκB-α were examined by Western blot. *β*-actin is shown as an internal control. (B) MDMs were stimulated with HIV and treated with either TREM1Ag (1 μg/mL) or U0126 (ERK inhibitor, 10 μM) and gene expression of IL-1β, IL-6, TNFα, INHBA and TREM1 are shown. (C) IL-1β and IL-6 secretion from MDMs treated as described in (B) levels were analyzed by ELISA. HIV stimulation was performed at a MOI = 1 for 24 hours. For qPCR, results are shown as fold induction compared to control samples after normalizing with 18S internal control. Data from repeated experiments were averaged and are expressed as means ± SD. *P≤0.05, **P≤0.01, ***P≤0.001; ns, non-significant.

After ligand binding, activation of TREM1 signaling is mediated by homotypic interactions between the immunoreceptor tyrosine-based activation motifs (ITAM) between TREM1 and the adaptor DNAX-Activation Protein 12 (DAP12). This interaction results in phosphorylation of DAP12 which facilitates the recruitment of Src family kinases to activate downstream signaling involving p38 mitogen-activated protein kinase (p38MAPK), c-Jun amino-terminal kinases (JNK) and the extracellular signal-regulated kinases1 and 2 (ERK1/2) pathways [28]. To determine which downstream signaling pathways are stimulated by TREM1 activation, selective inhibitors targeting ERK1/2, p38MAPK, or JNK were utilized after stimulating the TREM1 expressing THP1 cells with the TREM1 agonist. INHBA gene induction was assessed by qPCR analysis. Among them, U0126, an ERK1/2 inhibitor, significantly suppressed gene upregulation of INHBA (S8A Fig). We also performed Western blot analysis on MDMs following stimulation with HIV or HCV in the presence or absence of the TREM1 agonist. Fig 6A demonstrates that TREM1 activation resulted in increased levels of phosphorylated ERK1/2 following stimulation with HIV or HCV (Fig 6A and S8B Fig). Involvement of ERK signaling was further confirmed through studies utilizing U0126. In the presence of the inhibitor, the TREM1 agonist failed to enhance the expression of IL-1β, IL-6 and TNFα (Fig 6B and 6C, S8C and S8D Fig). In addition, we utilized ERK2 siRNA to knockdown and subsequently confirm the role of ERK in TREM1 signaling. In accordance with ERK1/2 inhibitor, silencing of ERK2 decreased inflammatory responses which were elicited by the TREM1 agonist (S9A and S9B Fig). Taken together, these findings support the conclusion that the robust activation of ERK signaling, following TREM1 stimulation, augments HIV or HCV driven inflammatory responses in macrophages.

### Inhibition of TREM1 signaling abrogates HIV-mediated inflammatory responses in macrophages

**Fig 7:**
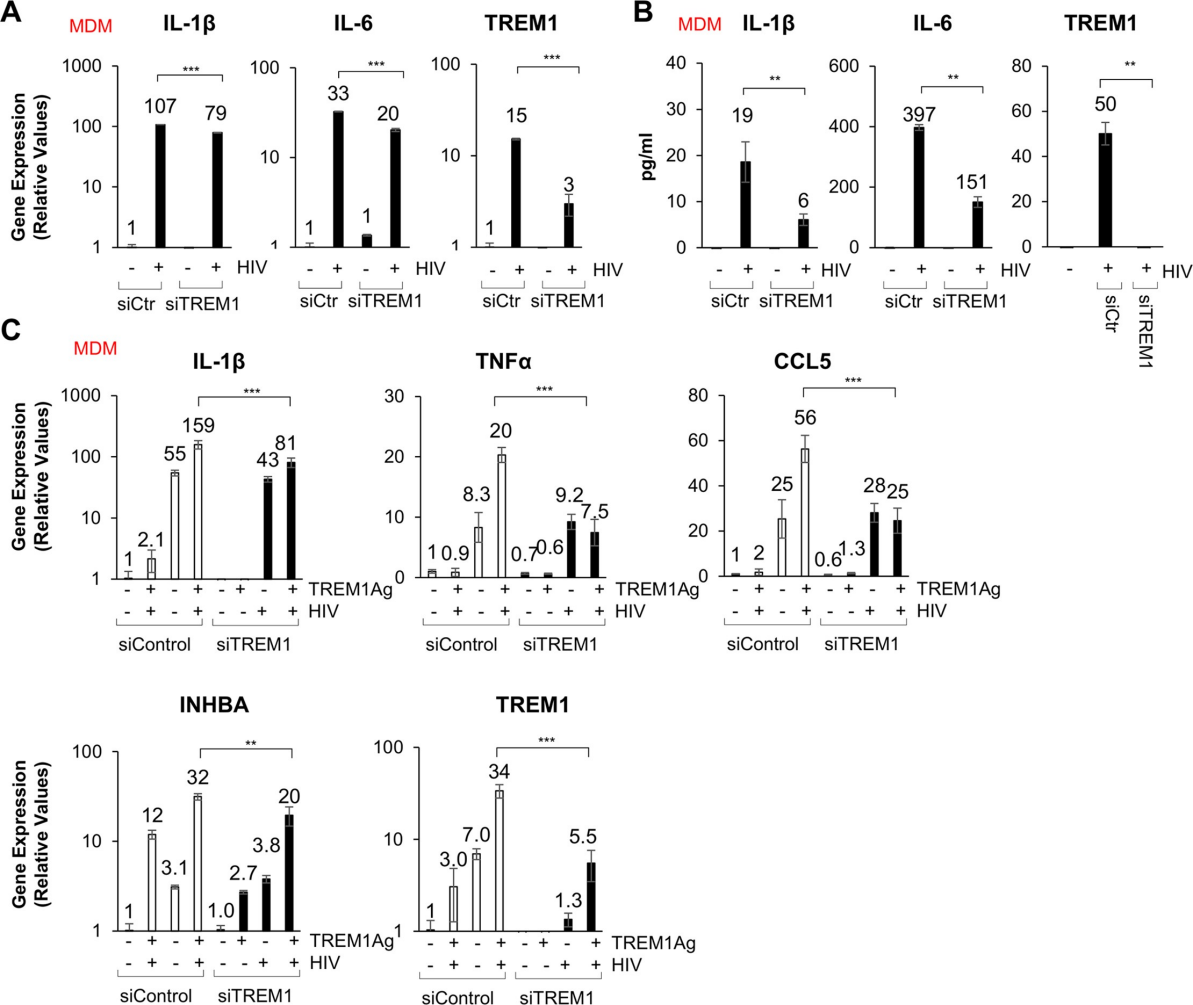
TREM1 inhibition decreases HIV mediated inflammatory cytokine production. (A) MDMs were treated with nonspecific control (siCtr) or TREM1 siRNA (siTREM1) for 48 hours and stimulated with HIV (MOI = 1 for 24 hours). Gene expression of IL-1β, IL-6, and TREM1 were analyzed. (B) Protein levels of IL-1β, IL-6 and TREM1 were measured by ELISA using supernatant of (A). (C) MDMs were treated as described in (A) and additionally stimulated with HIV ± TREM1Ag (1μg/mL). Gene expression of IL-1β, TNFα, CCL5, INHBA and TREM1 was performed by qPCR analysis. For qPCR, results are shown as fold induction compared to control samples after normalizing with 18S internal control. Data from repeated experiments were averaged and are expressed as means ± SD. *P≤0.05, **P≤0.01, ***P≤0.001; ns, non-significant.

To demonstrate that TREM1 signaling modulates HIV-induced inflammatory responses, we downregulated endogenous TREM1 expression using targeted siRNAs and confirmed siRNA knockdown of TREM1 protein expression by ELISA (Fig 7A and 7B). TREM1 was downregulated in MDMs by approximately 70% and the ability of HIV or HCV to upregulate inflammatory response was diminished in siRNA treated cells. In addition, HIV stimulation of IL-1β, IL-6, and TNFα was abrogated following downregulation of TREM1 in MDMs. Similarly, downregulation of TREM1 decreased IL-1β and IL-6 protein production in HCV stimulated MDMs (Fig 7A and 7B, and S10A and S10B Fig). The effect of TREM1 down-regulation on stimulation with the TREM1 agonist was also assessed. Our qPCR results demonstrated that TREM1 agonist mediated gene upregulation was decreased in cells treated with TREM1 siRNA (Fig 7C). Collectively, our data suggest that upregulation and subsequent activation of TREM1 modulates HIV or HCV induced inflammatory responses.

### TREM1 protein expression is increased in HIV infected individuals

**Fig 8:**
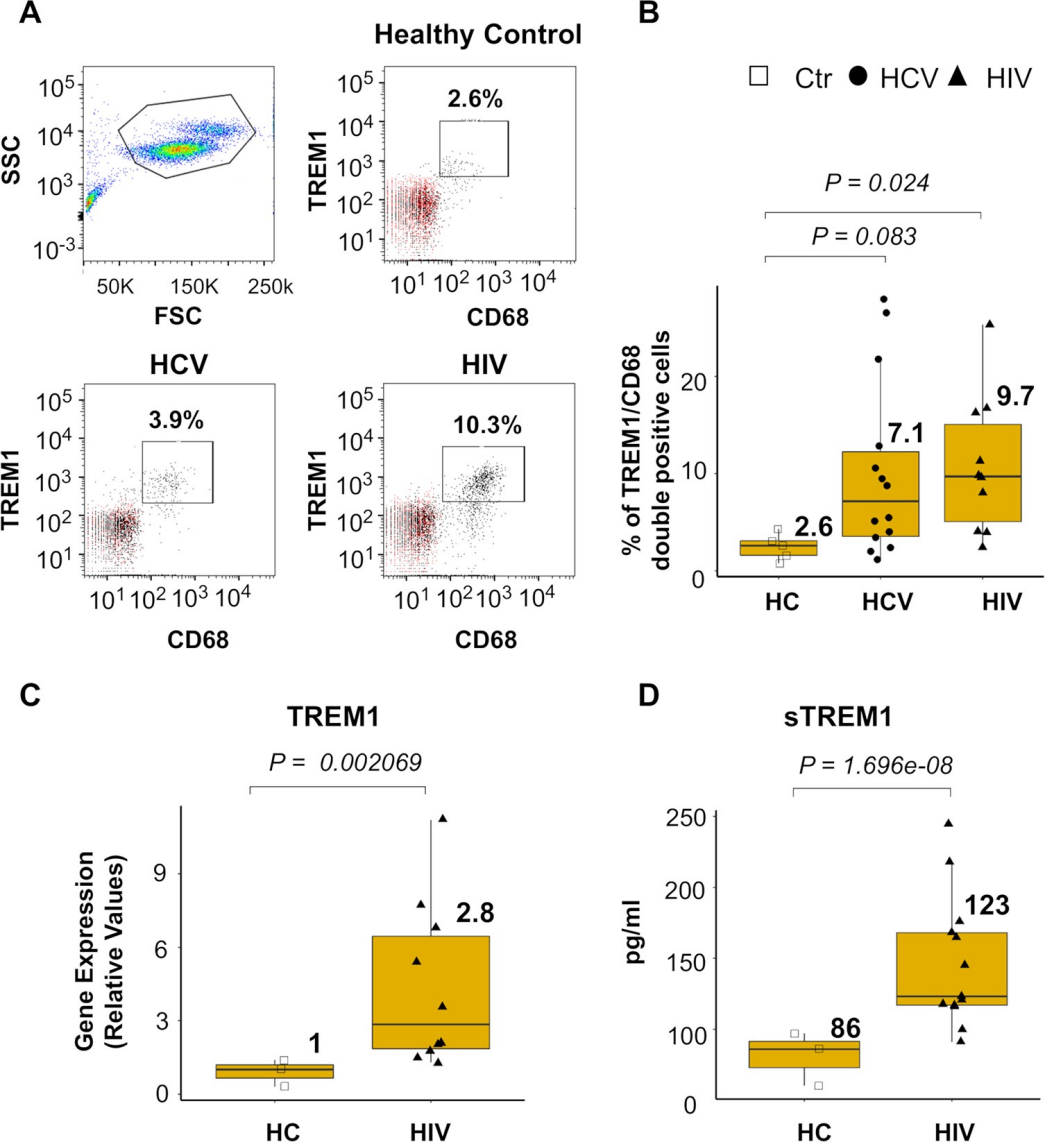
HIV infected individuals demonstrate significantly higher expression of TREM1 in PBMCs. (A) Peripheral blood mononuclear cells (PBMCs) samples from healthy controls (HC, n = 5), HCV infected (HCV, n = 14), or HIV infected (HIV, n = 10) patients. Flow cytometry analysis for TREM1 and CD68 was performed on all 3 groups and representative plots are shown. Red dot indicates the IgG control. (B) Box and whisker plots depicting the percentage of CD68 and TREM1 positive cells from all 3 patient groups. (C) Basal gene expression of TREM1 were examined in Healthy control (n = 3) and HIV infected individuals (n = 10) by qPCR analysis. (D) soluble TREM1(sTREM1) protein expression measured by ELISA in plasma from Healthy Controls (HC, n = 3) or HIV infected individuals (n = 13). For qPCR, results are shown as fold induction compared to control samples after normalizing with 18S internal control. The medians from the each group were compared using independent samples t-test from SPSS program. *P≤0.05, **P≤0.01, ***P≤0.001; ns, non-significant.

To validate our in vitro results, we obtained PBMC samples from HCV infected (n = 14), HIV infected (n = 10) and healthy individuals (n = 5) and the expression of TREM1 and CD68 was assessed by flow cytometry. We observed that the percentage of TREM1 positive macrophages in HCV-infected patients was not significantly different than the healthy control population. Importantly, HIV infected patients had a significantly higher number of TREM1 positive macrophages when compared to the uninfected controls (Fig 8A and 8B). Next, we further examined TREM1 expression in PBMCs and plasma from healthy controls (n = 3) and HIV infected individuals (n = 10). qPCR analysis demonstrated that PBMCs from HIV infected patients have significantly higher TREM1 expression levels when compared to healthy controls (Fig 8C). Similarly, plasma collected from HIV patients contained significantly higher levels of soluble (s)TREM1 (Fig 8D). Therefore, these results indicate that high TREM1 expression on myeloid cells, in the blood, may be a novel characteristic of HIV infected individuals and that targeting TREM1 signaling might provide a therapeutic strategy to ameliorate virus-induced chronic inflammation.

## Discussion

Despite effective control of AIDS by antiretroviral therapy (ART), the HIV pandemic is still a major health concern worldwide. Infected individuals frequently manifest chronic systemic inflammation that can result in cardiovascular, kidney and liver disease [38–41]. Similarly, HCV infection also causes chronic inflammation in the liver and ultimately the development of cirrhosis and HCC [7,9]. In this study, we characterized the innate immune response in KCs and other macrophages and elucidated a molecular mechanisms contributing to inflammatory responses observed in HIV or HCV infected individuals. We demonstrated that stimulation with HIV or HCV upregulates inflammatory cytokines, chemokines, and TREM1 expression which subsequently can amplify inflammatory responses. These inflammatory responses do not require viral infection or viral replication but are driven by exposure to viral proteins on intact virions. Similar to our studies, several papers have already demonstrated that HIV or HCV are capable of activating the inflammasome [32,33,42,43] which is another important driver of systemic inflammation. Specifically, our KC microarray data demonstrates that HIV activates antiviral and inflammatory responses and that these immune responses are elicited without viral infection and replication. The most upregulated subset of genes in KCs following HIV-1 exposure were antiviral genes. We observed increased levels of various interferon stimulated genes (ISGs) including IFI44L, ISG15, DDX58, RSAD2, IFITM and the IFIT family of proteins. Supporting this observation, previous reports have demonstrated that HIV stimulation leads to robust ISG and inflammatory gene upregulation in MDMs. Recently, Decalf et al. demonstrated that HIV fusion to the cell membrane of target cells induces low level IFN protein expression as determined by sensitive bead-based assays and secreted IFN ultimately contributes to the upregulation of ISGs [34]. However, in our studies, we demonstrated that endocytosis of HIV can also elicit inflammatory responses in macrophages but we were unable to demonstrate the production of IFN. Nasr et al. also investigated the mechanism of ISG induction by HIV in macrophages [44]. Their results demonstrated that at early time points, ISGs are upregulated by extracellular vesicles (EVs) within the viral inoculum that was used to stimulate macrophages. Our filtration experiments demonstrated that intact virions can also directly stimulate inflammatory responses in macrophages. In addition, they demonstrated that at later time points, newly synthesized mRNA from HIV can also stimulate inflammatory responses. However, the inflammatory response that we report here are independent of viral nucleic acids. Collectively, data from this report and others demonstrate that HIV stimulation leads to antiviral responses in macrophages and we have extended these studies to include specific regulation of TREM1 in KCs.

Importantly, our KC microarray analysis highlighted the activation of TREM1 signaling during HIV stimulation. TREM1 is exclusively expressed in the myeloid cell lineage and is involved in the amplification of production of inflammatory cytokines and chemokines during viral infection. We verified TREM1 upregulation by HIV and HCV in human macrophages including KCs and MDMs. We also demonstrated that cell surface recognition of the HIV particle through endocytosis stimulates TREM1 gene upregulation. Indeed, HIV viral proteins including gp120, pro, and rev, but not the HIV genome, trigger the upregulation of TREM1 gene expression. Moreover, experiments conducted with distinct cell signaling inhibitors revealed that the activation of NF-KappaB signaling is responsible for HIV mediated TREM1 gene upregulation. Importantly, we also observed significant upregulation of TREM1 in HIV infected patient samples. Supporting our observation, two recent studies have demonstrated that TREM1 upregulation occurs following stimulation with HIV viral proteins. Denner et al. examined the effect of the HIV-1 transmembrane envelope protein gp41 on PBMCs and characterized changes in gene expression of both proinflammatory cytokines and TREM1 through microarray analysis [36]. The authors also found increased levels of soluble TREM1 (sTREM1) in the supernatant from stimulated cells. sTREM1 is either a splice variant or it is a shed form of TREM1 and it has been considered a negative regulator of TREM1 [45]. We were able to detect it in HIV infected patient blood (n = 13, Fig 8D). Since PBMCs contain various cell types, it is possible that sTREM1 is produced by cells other than macrophages in response to gp41 stimulation [36]. Yuan et al. demonstrated that TREM1 is upregulated in MDMs following HIV stimulation. They also demonstrated that stimulation of Raw264.7 cells with recombinant gp120 and Tat proteins upregulated TREM1 expression and prevented cells from undergoing apoptosis. In our studies, we also found that gp160 and other HIV proteins upregulate TREM1, indicating that more than one HIV protein may induce TREM1 gene expression in vivo [37].

TREM1 was also upregulated on CD68 positive cells in the blood of HCV infected patients (Fig 8B) but the results were not significant. This may be due to the fact that unlike HIV, HCV is a hepatotrophic virus that does not cause significant hematologic pathology. Indeed, statistically significant results were obtained from the blood of HIV infected patients that demonstrated much greater expression on TREM1 (Fig 8). Future studies on hepatic expression of TREM1, using liver biopsies from infected patients, will be carried out to confirm our results obtained using KCs.

Studies focusing on other distinct viruses have also demonstrated that viral infection results in upregulation of TREM1 and that expression can be organ specific. Elevated expression of TREM1 was observed in the brains from mice infected with pathogenic West Nile virus (WNV) NY99 [46]. Marburg virus (MARV) and Ebola virus (EBOV) have also been reported to induce TREM1 gene expression in neutrophils [47]. sTREM1 is found in the sera of patients infected with Crimean Congo Haemorrhagic Fever (CCHF) virus, DENV, HBV, and HCV [48–51]. Accordingly, upregulation of TREM1 may modulate virus mediated inflammation as a general host defense mechanism and subsequent driver of disease progression in chronic viral infection. Kozik et al. has addressed the function of TREM1 by utilizing lymphocytic choriomeningitis virus (LCMV) that causes murine viral hepatitis in WT or TREM1 deficient mice. Their study revealed that LCMV infection upregulates TREM1 in neutrophils and that TREM1 deficiency decreased viral mediated liver damage. This indicates that TREM1 signaling can augment virus driven liver pathology as we propose for patients with HIV and HCV coinfection. Weber et al. demonstrated that TREM1 did not affect viral clearance but TREM1 deficiency increased the survival of mice during influenza virus infection [52]. Moreover, using an in vitro model, treatment with an antagonist peptide, LP17, dampened inflammatory responses and abrogated the production of TNF-α and IL-1β in MARV and EBOV stimulated neutrophils [47]. Our studies also demonstrated that activation of TREM1 signaling significantly enhanced HIV or HCV mediated inflammatory responses through the ERK signaling pathway. Given that TREM1 amplifies PRR signaling, it is likely that virus induced activation of PRRs in the liver [8,53] may also contribute to TREM1 associated hepatic inflammatory responses during HIV or HCV infection.

In the liver, TREM1 expression has been implicated in multiple hepatic diseases including liver cancer, fatty liver disease, alcoholic liver disease and other drivers of liver injury and fibrosis [54–57]. Duan et al. found that TREM1 not only modulates inflammation in liver tissue, but promotes the proliferation and spread of cancer cells. The authors demonstrated that higher expression of TREM1 in HCC patients contributed to mortality and increased recurrence [57,58]. Wu et al. has specifically demonstrated that TREM1 expression on KCs modulates inflammatory responses and drives the development of HCC using a diethylnitrosamine (DEN) induced HCC mouse model [30]. In addition, Nguyen-Lefebvre et al, from the same group, reported that KCs argument chronic liver inflammatory responses and promotes hepatic fibrogenesis through Notch and Oncostatin M (OSM) signaling using a carbon tetrachloride mouse model and the data was supported by analyzing samples from patients with hepatic fibrosis [57]. Perugorria et al reported that TREM2, a negative regulator of TREM1, is also upregulated on non-parenchymal liver cells in cirrhotic livers from humans and mice. The authors also reported that TREM2 deficiency heightened carbon tetrachloride and acetaminophen induced liver injury and that TREM2 carries out its protective functions by suppressing ROS production and lipid peroxidation [59]. These studies implicate TREM1, and associated regulatory molecules, in liver-specific inflammation and our data demonstrate that TREM1 upregulation by HIV and HCV, in macrophages, amplifies virus induced inflammatory responses. Additional studies have also reported that endogenous ligands for TREM1, HMGB1 and HSP70, are also upregulated in various liver diseases and may further drive hepatic inflammation through TREM1 [60–64]. This suggests that pharmacologic inhibition of TREM1, in patients with HIV and viral hepatitis coinfection, may be an attractive strategy to limit subsequent systemic and hepatic inflammation and the subsequent development of HCC.

## Materials and methods

### Cell culture

The human monocytic cell line THP-1 (ATCC) and H9 cells (ATCC) were maintained in RPMI 1640 medium (Invitrogen) containing 10% Fetal Bovine Serum (FBS) (45000–736, VWR) 1% penicillin/streptomycin (15140–122, Life Technologies), and 50mM β-Mercaptoethanol (21985023, ThermoFisher Scientific). Primary human monocytes were isolated by negative selection using the human monocyte enrichment kit (19059, Stem cell Technologies) from PBMCs which were isolated from the buffy coat by Ficoll-Paque Plus (17-1440-02, GE Healthcare) Then, monocytes were maintained Dulbecco’s Modified Eagle Medium containing 10% human serum (1830–0002, SeraCare), 1% L-glutamine (35050–061, Life Technologies), and 1% penicillin/streptomycin. Macrophage differentiation was conducted by culturing with 6 ng/ml M-CSF (300–25, Peprotech) for 4 days and maintained in fresh media without M-CSF for 2 or 3 days. Primary human Kupffer cells (KCs) were obtained from Life Technologies (HUKCCS) and maintained in Advanced DMEM (D5796, Sigma), 1% ITSx (41400–045, Thermo Fisher), 10% FBS, 15mM HEPES (15630–080, Life Technologies), 1% GlutaMax, and 1% penicillin/Streptomycin (Life Technologies). All cells were maintained at 37°C in 5% carbon dioxide.

### Study cohort

All participants were recruited from University of Miami, Jackson Memorial Hospital in Miami, FL. The three patient populations were volunteers in this study (a) 11 HIV infected, (b) 16 HCV infected, and (c) 5 healthy control. HIV or HCV infection was determined by OraQuick (1001–0079 and 1001–0181, OraSure) HIV or HCV antibody test. Peripheral blood mononuclear cells (PBMCs) were isolated and used for experiments. This study was approved by the Institutional Review Board at the University of Miami and Jackson Health System’s Office of Research and Grants.

### Ethics statement

This study was conducted using approved IRB by Coordinating Committee from University of Miami and University of Pittsburgh. All participants were adult and written informed consent was provided by the donors/ patients.

### Reagents

Alpha IFN 2α (IFN-α) was purchased from PBL (11105–1) and used to treat cells for 24 hours at 1000 U/ml unless otherwise indicated. Recombinant human IL-6 (200–06) and TNFα (300-01a) were purchased from Peprotech. polyinosinic-polycytidylic acid (poly(I:C)(tlrl-picw), 2’3’-cGAMP(tlrl-nacga23), LPS-B5 (tlrl-pb5lps) were purchased from Invivogen. IFN stimulatory DNA (ISD), which is a 90-bp non-CpG oligomer, was synthesized using following primers: 5’-TACAGATCTACTAGTGATCTATGACTGATCTGTACATGATCTACATACAGATCTACTAGTGATCTATGACTGATCTGTACATGATCTACA-3’ and 5’-TGTAGATCATGTACAGATCAGTCATAGATC ACTAGTAGATCTGTATGTAGATCATGTACAGATCAGTCATAGATCACTAGTAGATCTGTA-3’.

Signaling inhibitors used as following: Dynasore (ab120192, Abcam), BMS345541 (B9935, Sigma), BX795 (tlrl-bx7, Invivogen), SB203580 (S8307, Sigma), U0126 (Cell signaling), SB203580 (Sigma), and SP600125 (Sigma). All drugs were prepared in DMSO or water according to the company instruction. The following reagents were obtained through the NIH AIDS Reagent Program, Division of AIDS, NIAID, NIH: p96ZM651gp160-opt and p96ZM651gag-opt from Drs. Yingying Li, Feng Gao, and Beatrice H. Hahn (PMID: 14585212). Western blot analysis was performed using the following antibodies: anti-MDA5 (AT113, Enzo Life Sciences), anti-RIG-I (AT111, Enzo Life Sciences), anti-IFI16 (HPA002134, Sigma), anti-MAVS (ab31334, abcam), anti-IкBα (CL-21, Santa Cruz). All other antibodies are obtained from Cell Signaling: anti-MYD88 (4283), anti-STING (13647), anti-phospho ERK1/2(4370), anti-ERK1/2(4695). The following antibodies or agonists were used for neutralizing assay: anti-human TREM1 (Mab1278, R&D System), anti-human CD81 (555675, BD Biosciences), anti-human CD4 (344602, Biolegend), and Isotype Mouse IgG1 (400101, Biolegend). The following reagents were obtained through the NIH AIDS Reagent Program, Division of AIDS, NIAID, NIH: HIV-1 BaL gp120 Recombinant Protein (4961), HIV-1 HXB2 Rev Recombinant Protein (12707), HIV-1 HXB2 Pro Recombinant Protein (1178), and HIV-1 IIIB p24 Recombinant Protein (12028).

### Virus propagation, UV inactivation of virus, and viral genome isolation

Human embryonic kidney 293 cells (293T HEK cells, CRL-3216, ATCC) were transfected with HIV Bal encoding plasmid (provided by Dr. Stone) using Lipofectamine 3000(L3000-008, Life Technologies), and supernatant of the cells was collected and filtered through a 0.45-μM filter. Filtered supernatant was further concentrated using a 50KDa cut-off filter (VS15T32, Sartorius) after washing with PBS [65]. HIV IIIB strain were obtained from Dr. Dykxhoorn. HIV p24 amount was measured by ELISA (NEK050001KT, Perkin elmer) and capacity of viral replication was tested in TZM-BL reporter cells (NIH AIDS Reagent Program from Dr. John C. Kappes, Dr. Xiaoyun Wu and Tranzyme Inc) (100 ng p24 equivalents of HIV was calculated to be a MOI of 0.1. Unless otherwise stated, a MOI = 1 was used in the experiment. HCV JFH-1 was obtained from Dr. Jake Liang (NIH) and propagated as previously described [66]. For UV inactivation, HCV were UV irradiated in a UVC 500 UV Crosslinker (Hoefer) for a total dose of 0.6 J/cm^2^ and HIV was irradiated in a six-well cell culture dish at an intensity of 1.5 J/cm^2^ [67]. In order to isolate viral genome, virus containing supernatant was filtered through a 0.45-μM filter unit and precipitated using PEG-it Virus Concentration Solution (LV810A-1, System Biosciences). From the pellet, viral RNA isolation was performed using Trizol (15596–018, Life Technologies).

### Microarray analysis

After isolating the total RNA from MDMs with the RNeasy kit (74106, Qiagen), the isolated RNA was quantified with a NanoDrop (ThermoFisher) and was analyzed with an Agilent bioanalyzer (Agilent Technologies, Palo Alto, CA) for RNA quality. RNA was amplified with an Agilent Enzo kit and amplified complimentary RNA was subject to perform an Affymetrix Human 133 Plus 2.0 microarray chip containing 54,675 gene transcripts. Obtained data were normalized using the robust multiarray average (RMA) algorithm and analyzed by Partek Prosoftware (Partek, St. Charles, MO). To identify genes in the gene ontology analysis, we used the commercial gene pathway analysis web tool (https://portal.genego.com/). The signal values of each probe set ID from the selected gene lists were plotted by the commercial software Partek to generate the heat map.

Further pathway analysis was performed using Reactome Pathway Knowledgebase open-source software, a curated and peer-reviewed aggregate pathway database [68,69]. Results were supported by verification of Qiagen Ingenuity Pathway Analysis software. Gene set analysis was performed, and top ranked pathways by–log (p-value) were selected and visualized using Graphpad Prism 7 Software. The microarray data can be found using the Gene Expression Omnibus accession number GSE69589 (https://www.ncbi.nlm.nih.gov/genbank/).

### Quantitative real-time RT-PCR

Total RNA from cells were isolated with Trizol reagent (Invitrogen, USA) or RNAeasy kit (74106, Qiagen), and 1 μg of RNA was used for reverse-transcription to cDNA using qScript cDNA Supermix (101414–106, Quanta Bio-Sciences) according to manufacturer’s instructions. Real-time RT-PCR was performed using PerfeCTa FastMix II (97065–990, Quanta Bio-Sciences). Fluorescence real-time PCR reactions were run using C100 Touch Thermal Cycler (Bio-Rad) instrument. All primers and probes for quantitation of mRNA for target genes were purchased from IDT or ABI (Applied Biosystems) and FAM-Labeled TaqMan Probe, were normalized to endogenous control eukaryotic 18S ribosomal RNA. (4319413E, Life Technologies)

### siRNA gene knockdown in macrophages

Monocytes were cultured and differentiated in Upcell dish (Z688789, Sigma) and cells were detached cells with Accutase (561527, BD Bioscience). siControl NonTargeting siRNA (D-001210–02), Smart Pool siTREM1 (M-017974-00-005), Smart Pool siERK2 (M-003555-04-0005) were purchased from GE life science (Dharmacon). 0.25×10^6^ monocytes were differentiated to macrophages in a 48 well plate and transfected with small interfering RNA (siRNA) at a final concentration of 200nM/well using RNAiMAX (13778–030, Invitrogen). After 48 hours, cells were challenged with HIV or HCV and subjected to qPCR analysis.

### Flow cytometry

Differentiated Monocyte derived macrophages were treated with Accutase for 20 min and detached with gentle pipetting. Cells were washed with FACS buffer (5%FBS, 2mM EDTA, 0.09% sodium azide in PBS) and incubated with FcR binding inhibitor (14-9161-73, ebioscience) for 20min on ice. Cells were then incubated with human TREM-1 phycoerythrin conjugated monoclonal antibody (FAB1278P, R&D System), or mouse IgG1 phycoerythrin Isotype Control (IC002P, R&D System), 30min at 4°C. BD Cytoperm kit (BDB554714, BD Bioscience) was used for intracellular staining of CD68 using human CD68 BV421(BD564943, BD Bioscience) and data was acquired on a Becton Dickinson LSRF Fortessa (Beckman, USA) and analyzed with FlowJo (Tree Star, USA).

### ELISA

Supernatants from cells were harvested and ELISA was performed to detect Human CCL5 (DY278-05), IL-1β (DY1270-05), IL-6 (DY206-05), TREM1 (DTRM10C) according to the manufacture’s protocol. All ELISA kits were purchased from R&D System except VeriPlex Human Cytokine 16-Plex ELISA Kit (51510–1, PBL).

### Generation of TREM1 expressing stable cell lines

Human TREM1 encoding lentiviral plasmid (LV343336) and lentiviral packaging mix (LV003) were purchased from Applied Biological Materials Inc. 293T HEK cells were transfected with TREM1 expressing lentivirus production as it described in manufacture’s guideline. 1×10^6^ THP1 cells were used for lentiviral transduction with 1 μg/ml polybrene (H9268, Sigma). Twenty four hours post transduction, cells were washed with fresh media and maintained in selection media containing 2 μg/ml puromycin (A11138-03, Life Technologies). Expression level of TREM1 in THP1 cells (THP1-TREM1) was measured by ELISA and Flow cytometry.

### Statistical analysis

Statistics significance was determined by the unpaired student’s *t-test*. The asterisks indicate *p < 0.05, **p < 0.01, and ***p < 0.001.

## Supporting information

S1 Fig Comparison of pathogen recognition receptors (PRRs) expression in different macrophage cell types. (A) Basal gene expression of RIG-I, MDA-5, and MAVS were examined in THP1, KC, NPC, MDM, HRG, and PHH by qPCR analysis (n = 4). (B) Endogenous gene expression of TLR3, MYD88, and TRIF in different cell types were examined by qPCR analysis. (C) qPCR analysis of STING, cGAS, and IFI16 basal expression. (D) Protein levels of MDA-5, RIG-I, IFI16, MAVS, STING, and MYD88 were examined via Western blot in THP1 monocytes, MDMs, and NPCs ± IFNα (1000 U/ml). *β*-actin was used as internal control. For qPCR, results are shown as fold induction compared to control samples after normalizing with 18S internal control. Data are from one experiment. *P≤0.05, **P≤0.01, ***P≤0.001; ns, non-significant.
(TIF)

S2 Fig The HIV virion induces inflammatory responses in macrophages. (A) qPCR analysis of antiviral gene expression from KCs following stimulation with IFNα (10 U/mL) or increasing MOIs of HIV (1, 10). (B) qPCR analysis of gene expression of CXCL10, and TREM1 in KCs, NPCs, and MDMs after directly incubating virus filtrates, and pNL-Bal plasmid(0.1ug/ml), HIV (MOI = 1), and HCV(MOI = 1). (C) Flow cytometry analysis of MDMs after staining with anti-CD68, anti-CD15, and anti-CD209 antibodies. For qPCR, results are shown as fold induction compared to control samples after normalizing with 18S internal control. Data from repeated experiments were averaged and are expressed as means ± SD. *P≤0.05, **P≤0.01, ***P≤0.001; ns, non-significant.
(TIF)

S3 Fig HIV stimulation induces inflammatory gene expression in KCs and MDMs independent of virus replication. (A) qPCR analysis of gene expression of IL-1β, IL-6, TREM1, and CCL5 after treating MDMs with LPS (10ug/ml) or HIV (MOI = 1 or 10) for 24 hours. (B) qPCR analysis of gene expression kinetics of IL-1β, IL-6, TREM1, and CCL5 in HIV exposed KC at an MOI of 1 at 3, 8, or 24 hours. (C) MDMs were treated with HIV entry neutralizing antibody CD4 antibody (10 μg/ml) or control IgG isotype (10 μg/ml) and subsequently exposed to HIV (MOI = 1) for 24 hours. Gene expression of CCL5, IL-6, TREM1, and IL-1β were analyzed by qPCR. (D) HIV or UV irradiated HIV (MOI = 1) were incubated with MDM for 24 hours and qPCR analysis was conducted using total RNA from the MDM. Gene expression levels of CCL5, IL-6, TREM1, and IL-1β are shown. (E) p24 Staining of MDMs after stimulation with HIV or UV irradiated HIV for 1 hour. (F) TZM-bl cells were infected with HIV or UV irradiated HIV (MOI = 1, 48 hours) and analyzed for viral replication. Replication inhibitor Zidovudine (AZT, 25uM) used as a positive control. For qPCR, results are shown as fold induction compared to control samples after normalizing with 18S internal control. Data from repeated experiments were averaged and are expressed as means ± SD. *P≤0.05, **P≤0.01, ***P≤0.001; ns, non-significant.
(TIF)

S4 Fig HCV induces inflammatory immune responses in KC. (A) KCs were stimulated with IFNα (10 U/mL) or increasing MOI of HCV (1, 10, 100) and qPCR analysis of IL-1β, IL-6, TREM1, CXCL10, CCL5, and IL-10 are shown. (B) ELISA of IL-1β, IL-6, and CCL5 levels in supernatants of KCs treated with HCV at a MOI = 1 or 10. (C) Flow cytometry analysis of TREM1 surface expression in MDMs after exposure of HCV (MOI = 1) for 2 or 6 days. For qPCR, results are shown as fold induction compared to control samples after normalizing with 18S internal control. Data from repeated experiments were averaged and are expressed as means ± SD. *P≤0.05, **P≤0.01, ***P≤0.001; ns, non-significant.
(TIF)

S5 Fig HCV replication is dispensable for inflammatory genes expression in KCs and MDMs. (A) qPCR result of gene expression of IL-1β, IL-6, TREM1, and CCL5 after treating MDMs with LPS (10ug/ml) or HCV (MOI = 1 or 10) for 24 hours. (B) qPCR analysis of gene expression kinetics of IL-1β, IL-6, TREM1, and CCL5 in HCV exposed KC at an MOI of 1 at 3, 8, or 24 hours. (C) MDMs were pretreated with HCV entry neutralizing antibody CD81 (10 μg/ml) or control IgG isotype (10 μg/ml) and the cells were subsequently exposed to HIV (MOI = 1) for 24 hours. Gene expression of CCL5, IL-6, TREM1, and IL-1β were analyzed by qPCR. (D) MDMs were incubated with HCV or UV irradiated HCV (MOI = 1) for 24 hours and qPCR analysis was conducted using total RNA from the MDMs. Gene expression levels of CCL5, IL-6, TREM1, and IL-1β. (E) Huh 7.5.1 cells were infected with different MOI of HCV or UV irradiated HCV(MOI = 0.1, 1). After 5 days of infection, HCV core protein expression was examined using Western blot analysis. For qPCR, results are shown as fold induction compared to control samples after normalizing with 18S internal control. Data from repeated experiments were averaged and are expressed as means ± SD. *P≤0.05, **P≤0.01, ***P≤0.001; ns, non-significant.
(TIF)

S6 Fig Endocytosis of HCV stimulates TREM1 gene induction. (A) MDMs were stimulated with HCV (MOI = 1) ± Dynasore (80 μM) for 24 hours and TREM1 gene expression was analyzed by qPCR. (B) qPCR result of TREM1 gene expression after treating MDMs with supernatants from stimulated MDMs for 3 or 24 hours. (C) MDMs were treated with TNFα (20 ng/ml), IL-6 (10 ng/ml), and IFNα (1000 U/mL) for 3 and 24 hours and TREM1 gene expression was examined by qPCR. (D) MDMs were transfected (t) with poly(I:C) (2 μg/ml), ISD (2 μg/ml) or 2’3GAMP (2 μg/ml) for 24 hours and gene expression of TREM1 and CXCL10 were examined by qPCR. (E) qPCR analysis of TREM1 in NPCs after directly incubating 20 μg of HIV or HCV genomic RNA for the indicated time points. (F) TREM1 gene expression following treatment with cell signaling inhibitors in MDMs. BMS345541 (IKK inhibitor, 2 μM), BX795 (TBK1 inhibitor, 5 μM), SB203580 (MAPK inhibitor, 20 μM) were used. (G) Cell viability was assessed by Alamar blue reduction assay for the chemical inhibitors used. Percentage reduction of AlamarBlue reagent was calculated based on manufacturer guidelines. For qPCR, results are shown as fold induction compared to control samples after normalizing with 18S internal control. Data from repeated experiments were averaged and are expressed as means ± SD. *P≤0.05, **P≤0.01, ***P≤0.001; ns, non-significant.
(TIF)

S7 Fig Treatment with a specific TREM1 agonist increases HCV mediated inflammatory responses. (A) Flow cytometry analysis of TREM1 surface expression on THP1-TREM1 cells which were generated using TREM1 encoding lentivirus transduction. (B) qPCR analysis was used to examine basal gene expression of TREM1, NFKBA1, IKBKB, MYD88, and TRIF in THP1 or THP1-TREM1 cells. (C) THP1 or THP1-TREM1 cells were placed on a precoated plate containing isotype IgG or TREM1 agonist (1ug/ml) and INBHA gene expression was analyzed by qPCR. (D) MDMs were treated with IgG control or TREM1 agonist and subsequently stimulated with HCV (MOI = 1) for 24 hours. Gene expression of INHBA, TREM1, IL-1β, IL-6, and TNFα were measured by qPCR analysis. (E, F) Supernatants of MDMs or NPCs were analyzed for IL-1β and IL-6 levels by ELISA. Cells were stimulated with HCV (MOI = 1, 24 hours) with or without TREM1 agonist (1ug/ml). For qPCR, results are shown as fold induction compared to control samples after normalizing with 18S internal control. Data from repeated experiments were averaged and are expressed as means ± SD. *P≤0.05, **P≤0.01, ***P≤0.001; ns, non-significant.
(TIF)

S8 Fig The ERK pathway controls TREM1 activation and is involved in HCV inflammatory responses. (A) THP1-TREM1 overexpressing cells were treated with the TREM1 agonist (1 μg/mL) ± mock, U0126 (ERK inhibitor, 10 μM), SB203580 (p38 MAPK inhibitor, 10 μM), or SP600125 (JNK inhibitor, 20 μM). Gene expression of INHBA was analyzed by qPCR. (B) MDMs were stimulated with either mock or HCV ± TREM1Ag and protein expression levels of phospho-ERK, total ERK, and IκB-α were analyzed by Western blot. *β*-actin was used as an internal control. (C) MDMs were stimulated with HCV ± TREM1Ag ± U0126 and gene expression of INHBA, TREM1, and IL-1β were analyzed by qPCR. (D) MDMs were treated as in (C) and IL-1β and IL-6 levels in the supernatant were measured by ELISA. All treatments were performed for 24 hours at a MOI. = 1. For qPCR, results are shown as fold induction compared to control samples after normalizing with 18S internal control. Data from repeated experiments were averaged and are expressed as means ± SD. *P≤0.05, **P≤0.01, ***P≤0.001; ns, non-significant.
(TIF)

S9 Fig Knockdown of ERK2 dampens TREM1 augmented inflammatory responses. (A) Gene expression of IL-1β, IL-6, TNFα, TREM1, and INHBA in MDMs after silencing with nonspecific control or ERK2 siRNA and stimulation with HIV in the absence or presence of the TREM1 agonist (1ug/ml). Western blot analysis of indicated proteins from MDMs transfected (t) with non-specific (siCtr) or ERK2 siRNA (siERK2). (B) Gene expression of IL-1β, IL-6, TNFα, TREM1, and INHBA in MDMs after silencing with nonspecific control or ERK2 siRNA and stimulation with HCV in the absence or presence of the TREM1 agonist (1ug/ml). For qPCR, results are shown as fold induction compared to control samples after normalizing with 18S internal control. Data from repeated experiments were averaged and are expressed as means ± SD. *P≤0.05, **P≤0.01, ***P≤0.001; ns, non-significant.
(TIF)

S10 Fig Blocking TREM1 activity downregulates inflammatory response in MDMs following HCV stimulation. (A) MDMs were treated with control or TREM1 siRNA for 48 hours. Cells were then stimulated with HCV for 24 hours and gene expression of IL-1β, IL-6, and TREM1 were analyzed by qPCR. (B) MDMs were treated as described in (A) and supernatants were analyzed for secretion of IL-1β and IL-6 by ELISA assay. For qPCR, results are shown as fold induction compared to control samples after normalizing with 18S internal control. Data from repeated experiments were averaged and are expressed as means ± SD. *P≤0.05, **P≤0.01, ***P≤0.001; ns, non-significant.
(TIF)

S11 Fig Proposed model of the role of TREM1 in HIV/HCV infected patients. HIV and HCV or viral proteins are internalized by Kupffer or myeloid cells, through dynamin dependent endocytosis, and stimulate upregulation of proinflammatory cytokines and chemokines. These viruses drive upregulation TREM1 expression on the cell surface through NF-кB signaling. Engagement of the TREM1 receptor with its putative ligands, including HMGB1 and HSP70, further activate the ERK1/2 signaling pathway leading to subsequent inflammatory cytokine production.
(TIF)
