# Supplementary figures and images for: HIV and HCV augments inflammatory responses through increased TREM-1 expression and signaling in Kupffer and Myeloid cells
Source: PLoS Pathog. 2019 Jul 1;15(7):e1007883. doi: 10.1371/journal.ppat.1007883 (PMC6625740; doi:10.1371/journal.ppat.1007883)

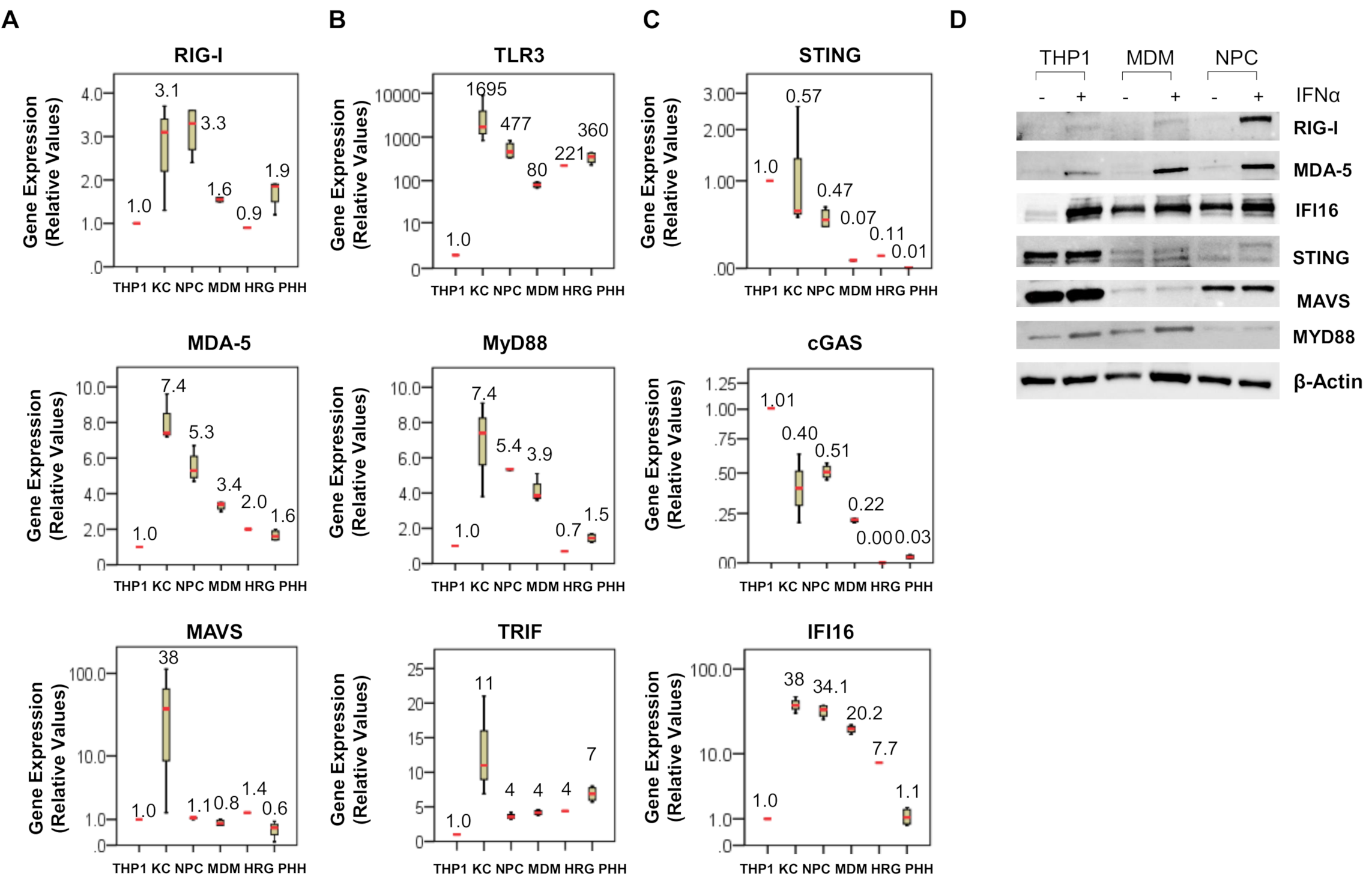

Supplement: S1 Fig — (A) Basal gene expression of RIG-I, MDA-5, and MAVS were examined in THP1, KC, NPC, MDM, HRG, and PHH by qPCR analysis (n = 4). (B) Endogenous gene expression of TLR3, MYD88, and TRIF in different cell types were examined by qPCR analysis. (C) qPCR analysis of STING, cGAS, and IFI16 basal expression. (D) Protein levels of MDA-5, RIG-I, IFI16, MAVS, STING, and MYD88 were examined via Western blot in THP1 monocytes, MDMs, and NPCs ± IFNα (1000 U/ml). β-actin was used as internal control. For qPCR, results are shown as fold induction compared to control samples after normalizing with 18S internal control. Data are from one experiment. *P≤0.05, **P≤0.01, ***P≤0.001; ns, non-significant. (TIF) [file ppat.1007883.s001.tif]

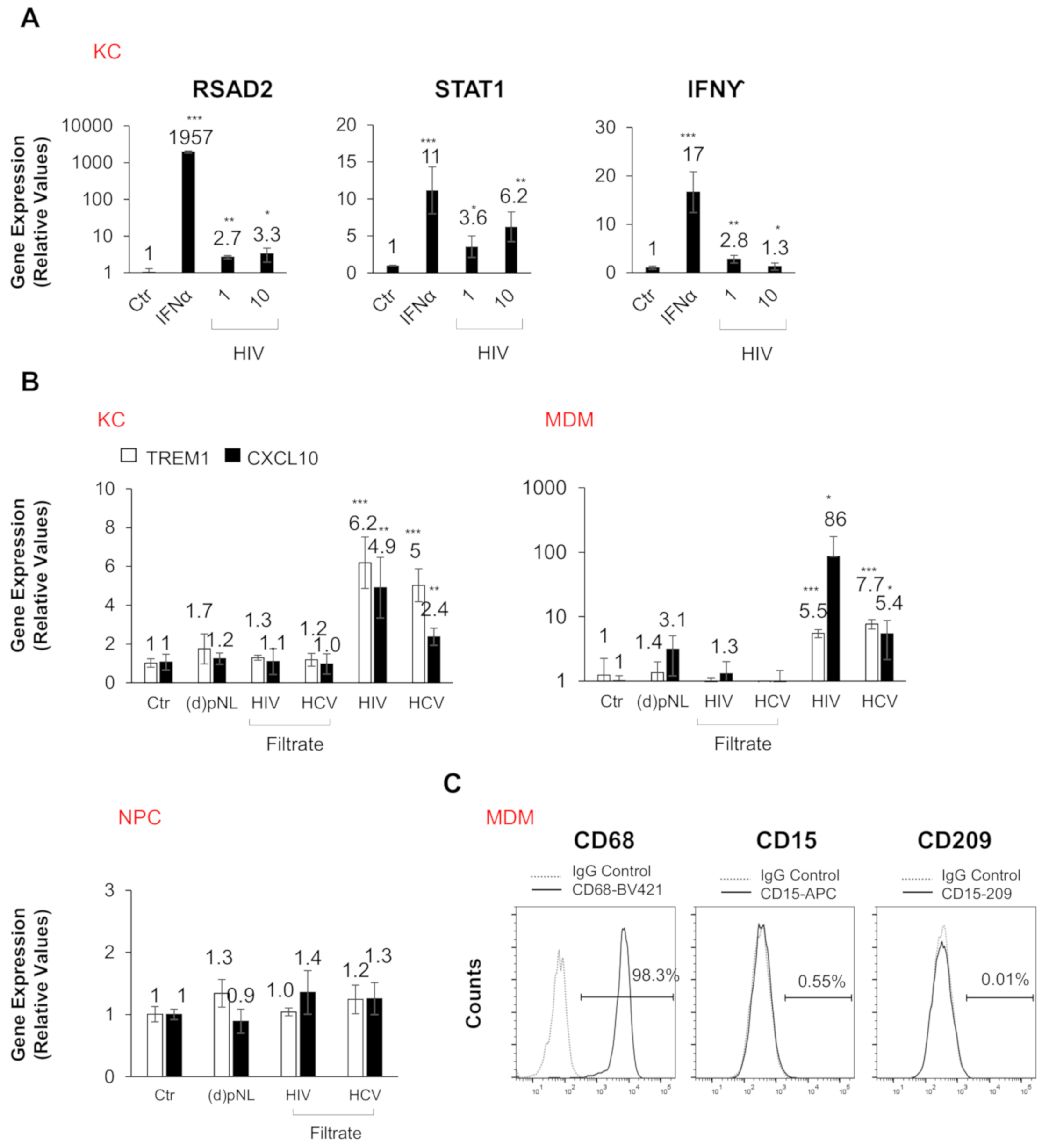

Supplement: S2 Fig — (A) qPCR analysis of antiviral gene expression from KCs following stimulation with IFNα (10 U/mL) or increasing MOIs of HIV (1, 10). (B) qPCR analysis of gene expression of CXCL10, and TREM1 in KCs, NPCs, and MDMs after directly incubating virus filtrates, and pNL-Bal plasmid(0.1ug/ml), HIV (MOI = 1), and HCV(MOI = 1). (C) Flow cytometry analysis of MDMs after staining with anti-CD68, anti-CD15, and anti-CD209 antibodies. For qPCR, results are shown as fold induction compared to control samples after normalizing with 18S internal control. Data from repeated experiments were averaged and are expressed as means ± SD. *P≤0.05, **P≤0.01, ***P≤0.001; ns, non-significant. (TIF) [file ppat.1007883.s002.tif]

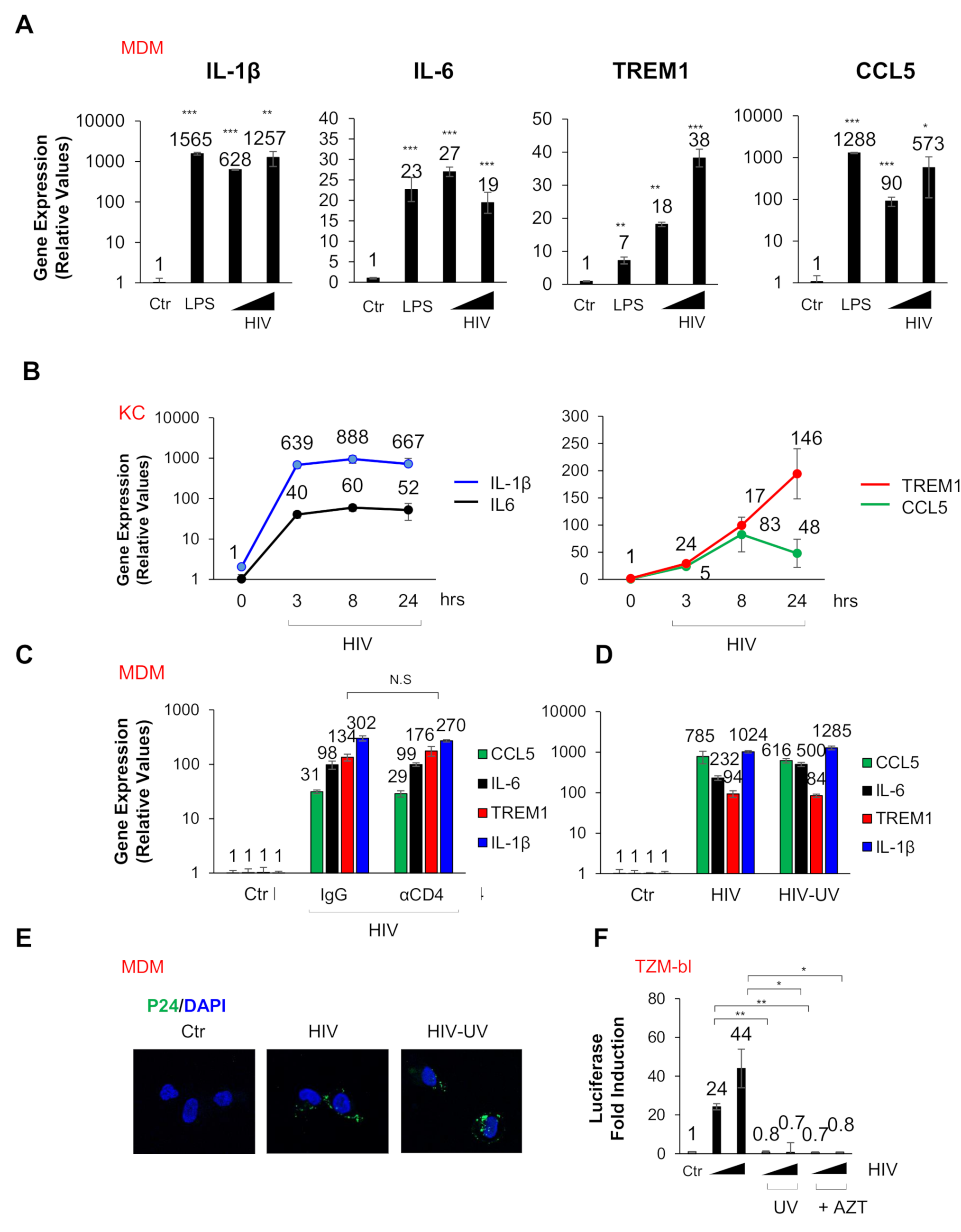

Supplement: S3 Fig — (A) qPCR analysis of gene expression of IL-1β, IL-6, TREM1, and CCL5 after treating MDMs with LPS (10ug/ml) or HIV (MOI = 1 or 10) for 24 hours. (B) qPCR analysis of gene expression kinetics of IL-1β, IL-6, TREM1, and CCL5 in HIV exposed KC at an MOI of 1 at 3, 8, or 24 hours. (C) MDMs were treated with HIV entry neutralizing antibody CD4 antibody (10 μg/ml) or control IgG isotype (10 μg/ml) and subsequently exposed to HIV (MOI = 1) for 24 hours. Gene expression of CCL5, IL-6, TREM1, and IL-1β were analyzed by qPCR. (D) HIV or UV irradiated HIV (MOI = 1) were incubated with MDM for 24 hours and qPCR analysis was conducted using total RNA from the MDM. Gene expression levels of CCL5, IL-6, TREM1, and IL-1β are shown. (E) p24 Staining of MDMs after stimulation with HIV or UV irradiated HIV for 1 hour. (F) TZM-bl cells were infected with HIV or UV irradiated HIV (MOI = 1, 48 hours) and analyzed for viral replication. Replication inhibitor Zidovudine (AZT, 25uM) used as a positive control. For qPCR, results are shown as fold induction compared to control samples after normalizing with 18S internal control. Data from repeated experiments were averaged and are expressed as means ± SD. *P≤0.05, **P≤0.01, ***P≤0.001; ns, non-significant. (TIF) [file ppat.1007883.s003.tif]

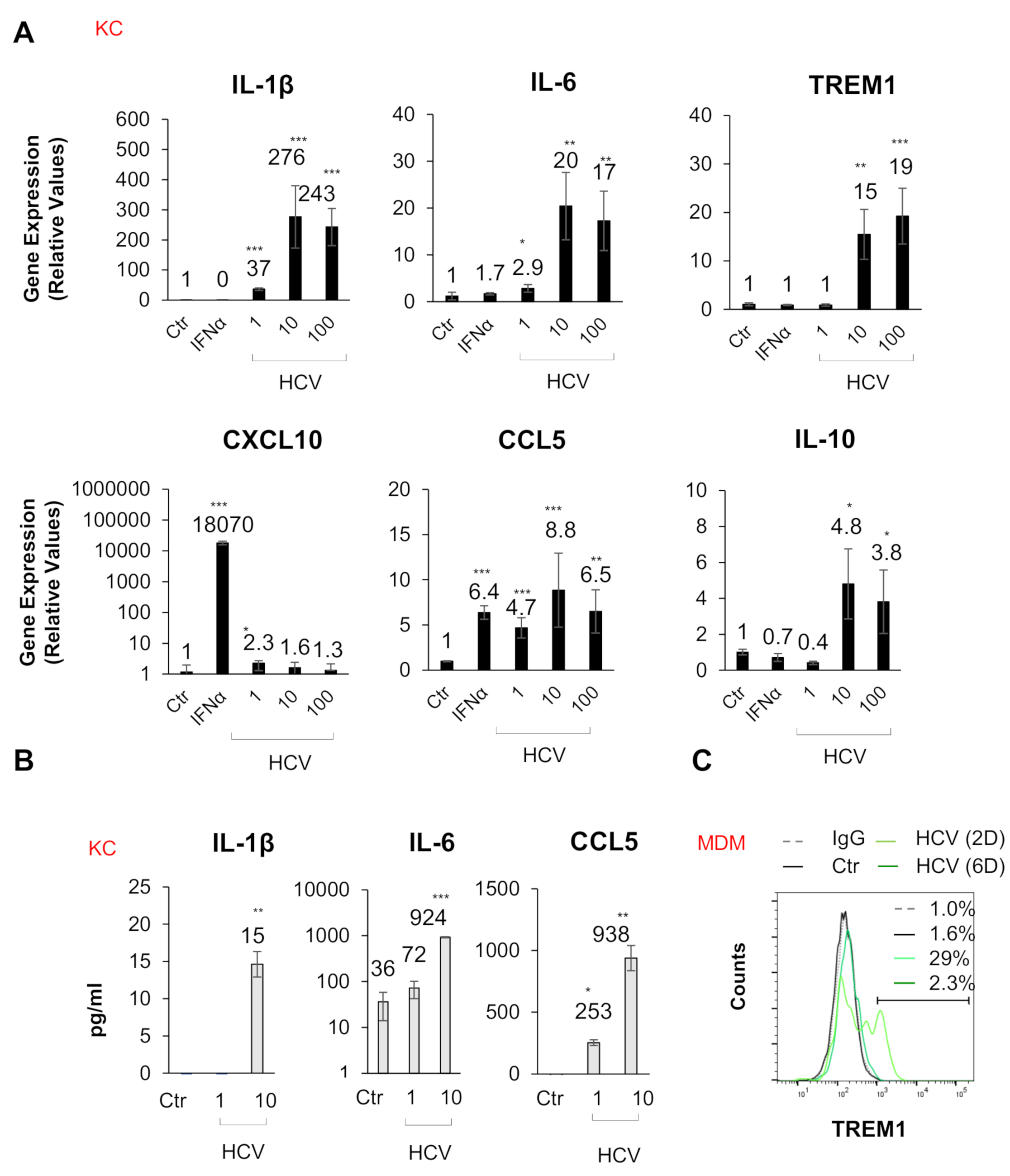

Supplement: S4 Fig — (A) KCs were stimulated with IFNα (10 U/mL) or increasing MOI of HCV (1, 10, 100) and qPCR analysis of IL-1β, IL-6, TREM1, CXCL10, CCL5, and IL-10 are shown. (B) ELISA of IL-1β, IL-6, and CCL5 levels in supernatants of KCs treated with HCV at a MOI = 1 or 10. (C) Flow cytometry analysis of TREM1 surface expression in MDMs after exposure of HCV (MOI = 1) for 2 or 6 days. For qPCR, results are shown as fold induction compared to control samples after normalizing with 18S internal control. Data from repeated experiments were averaged and are expressed as means ± SD. *P≤0.05, **P≤0.01, ***P≤0.001; ns, non-significant. (TIF) [file ppat.1007883.s004.tif]

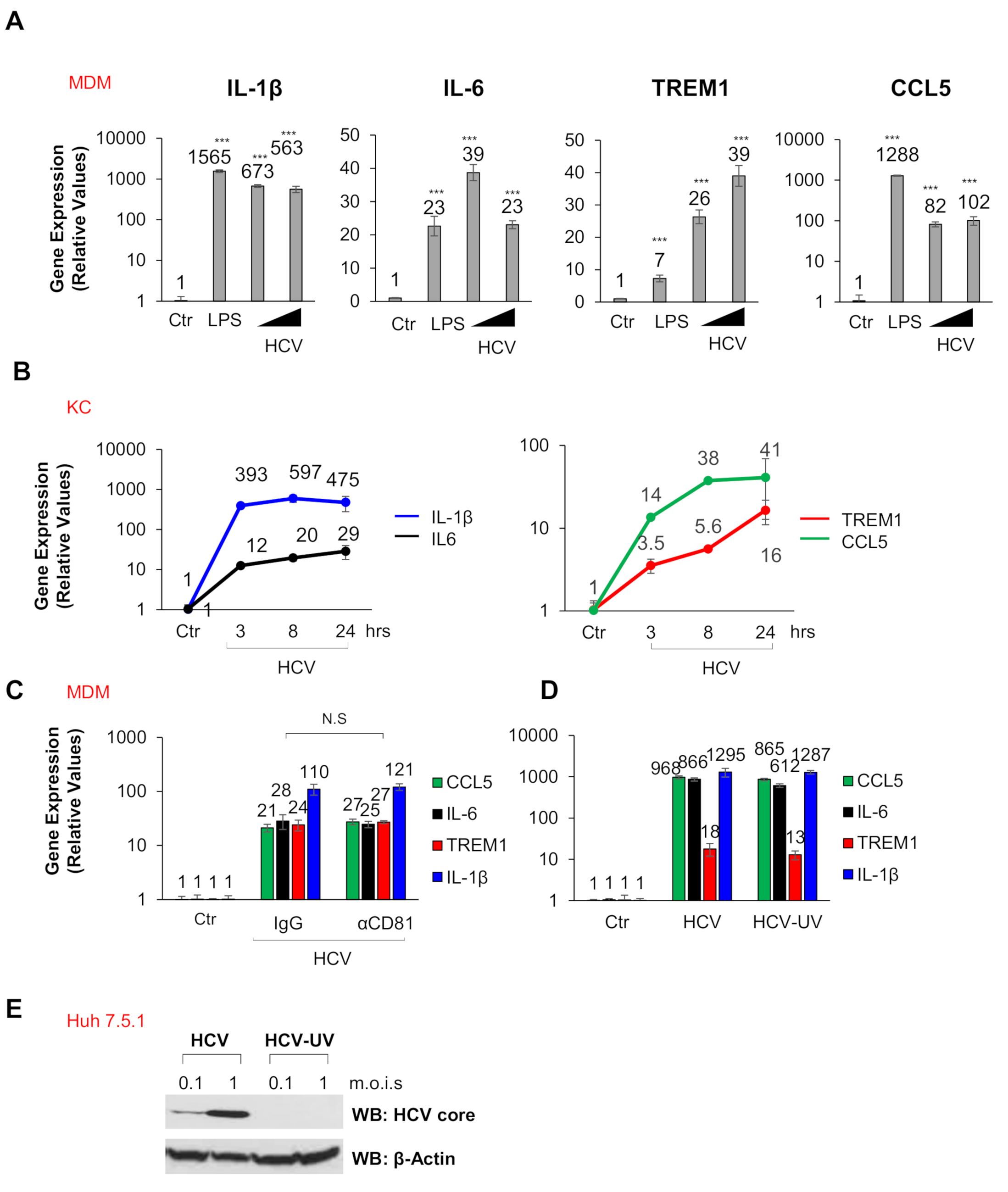

Supplement: S5 Fig — (A) qPCR result of gene expression of IL-1β, IL-6, TREM1, and CCL5 after treating MDMs with LPS (10ug/ml) or HCV (MOI = 1 or 10) for 24 hours. (B) qPCR analysis of gene expression kinetics of IL-1β, IL-6, TREM1, and CCL5 in HCV exposed KC at an MOI of 1 at 3, 8, or 24 hours. (C) MDMs were pretreated with HCV entry neutralizing antibody CD81 (10 μg/ml) or control IgG isotype (10 μg/ml) and the cells were subsequently exposed to HIV (MOI = 1) for 24 hours. Gene expression of CCL5, IL-6, TREM1, and IL-1β were analyzed by qPCR. (D) MDMs were incubated with HCV or UV irradiated HCV (MOI = 1) for 24 hours and qPCR analysis was conducted using total RNA from the MDMs. Gene expression levels of CCL5, IL-6, TREM1, and IL-1β. (E) Huh 7.5.1 cells were infected with different MOI of HCV or UV irradiated HCV(MOI = 0.1, 1). After 5 days of infection, HCV core protein expression was examined using Western blot analysis. For qPCR, results are shown as fold induction compared to control samples after normalizing with 18S internal control. Data from repeated experiments were averaged and are expressed as means ± SD. *P≤0.05, **P≤0.01, ***P≤0.001; ns, non-significant. (TIF) [file ppat.1007883.s005.tif]

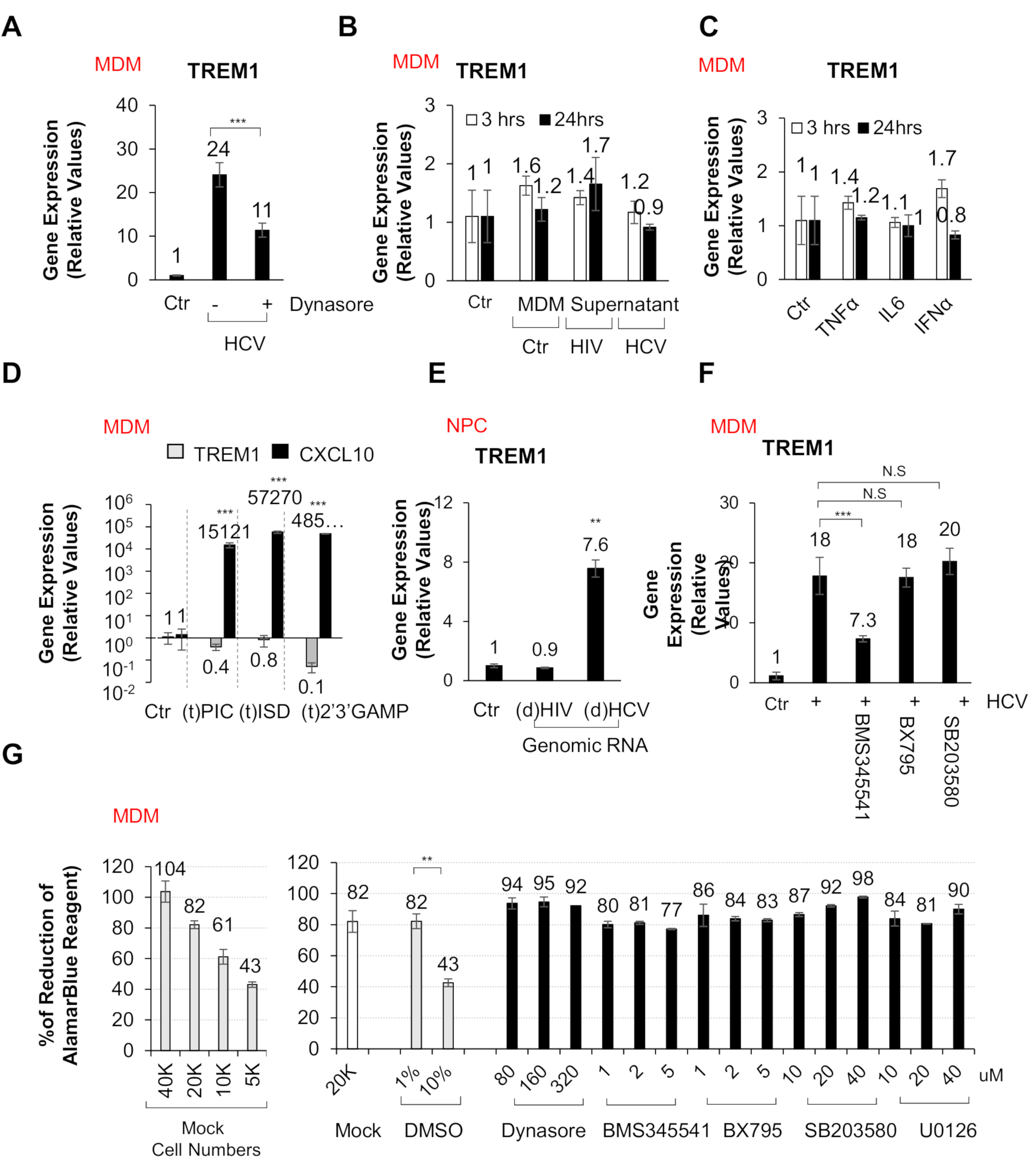

Supplement: S6 Fig — (A) MDMs were stimulated with HCV (MOI = 1) ± Dynasore (80 μM) for 24 hours and TREM1 gene expression was analyzed by qPCR. (B) qPCR result of TREM1 gene expression after treating MDMs with supernatants from stimulated MDMs for 3 or 24 hours. (C) MDMs were treated with TNFα (20 ng/ml), IL-6 (10 ng/ml), and IFNα (1000 U/mL) for 3 and 24 hours and TREM1 gene expression was examined by qPCR. (D) MDMs were transfected (t) with poly(I:C) (2 μg/ml), ISD (2 μg/ml) or 2’3GAMP (2 μg/ml) for 24 hours and gene expression of TREM1 and CXCL10 were examined by qPCR. (E) qPCR analysis of TREM1 in NPCs after directly incubating 20 μg of HIV or HCV genomic RNA for the indicated time points. (F) TREM1 gene expression following treatment with cell signaling inhibitors in MDMs. BMS345541 (IKK inhibitor, 2 μM), BX795 (TBK1 inhibitor, 5 μM), SB203580 (MAPK inhibitor, 20 μM) were used. (G) Cell viability was assessed by Alamar blue reduction assay for the chemical inhibitors used. Percentage reduction of AlamarBlue reagent was calculated based on manufacturer guidelines. For qPCR, results are shown as fold induction compared to control samples after normalizing with 18S internal control. Data from repeated experiments were averaged and are expressed as means ± SD. *P≤0.05, **P≤0.01, ***P≤0.001; ns, non-significant. (TIF) [file ppat.1007883.s006.tif]

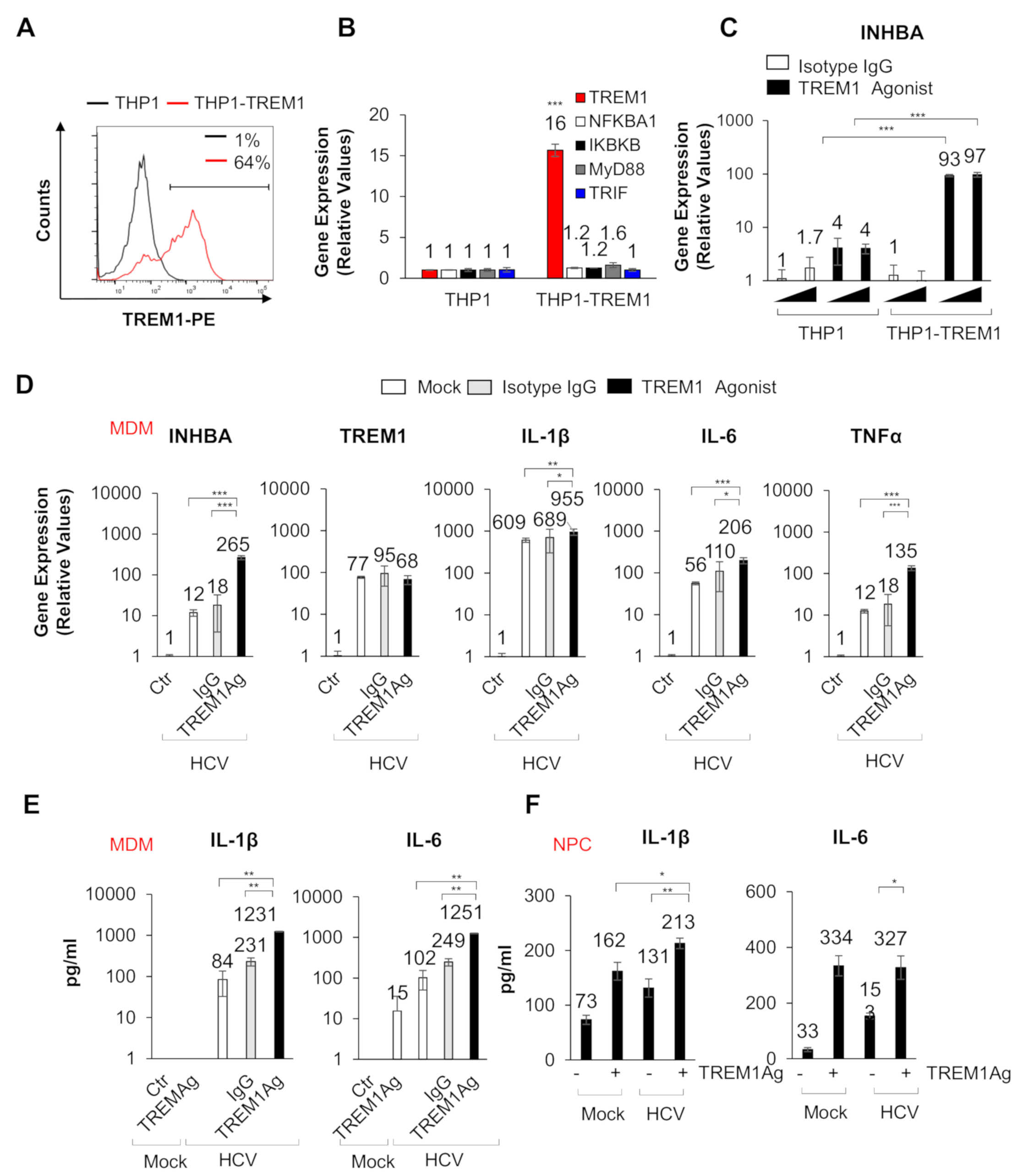

Supplement: S7 Fig — (A) Flow cytometry analysis of TREM1 surface expression on THP1-TREM1 cells which were generated using TREM1 encoding lentivirus transduction. (B) qPCR analysis was used to examine basal gene expression of TREM1, NFKBA1, IKBKB, MYD88, and TRIF in THP1 or THP1-TREM1 cells. (C) THP1 or THP1-TREM1 cells were placed on a precoated plate containing isotype IgG or TREM1 agonist (1ug/ml) and INBHA gene expression was analyzed by qPCR. (D) MDMs were treated with IgG control or TREM1 agonist and subsequently stimulated with HCV (MOI = 1) for 24 hours. Gene expression of INHBA, TREM1, IL-1β, IL-6, and TNFα were measured by qPCR analysis. (E, F) Supernatants of MDMs or NPCs were analyzed for IL-1β and IL-6 levels by ELISA. Cells were stimulated with HCV (MOI = 1, 24 hours) with or without TREM1 agonist (1ug/ml). For qPCR, results are shown as fold induction compared to control samples after normalizing with 18S internal control. Data from repeated experiments were averaged and are expressed as means ± SD. *P≤0.05, **P≤0.01, ***P≤0.001; ns, non-significant. (TIF) [file ppat.1007883.s007.tif]

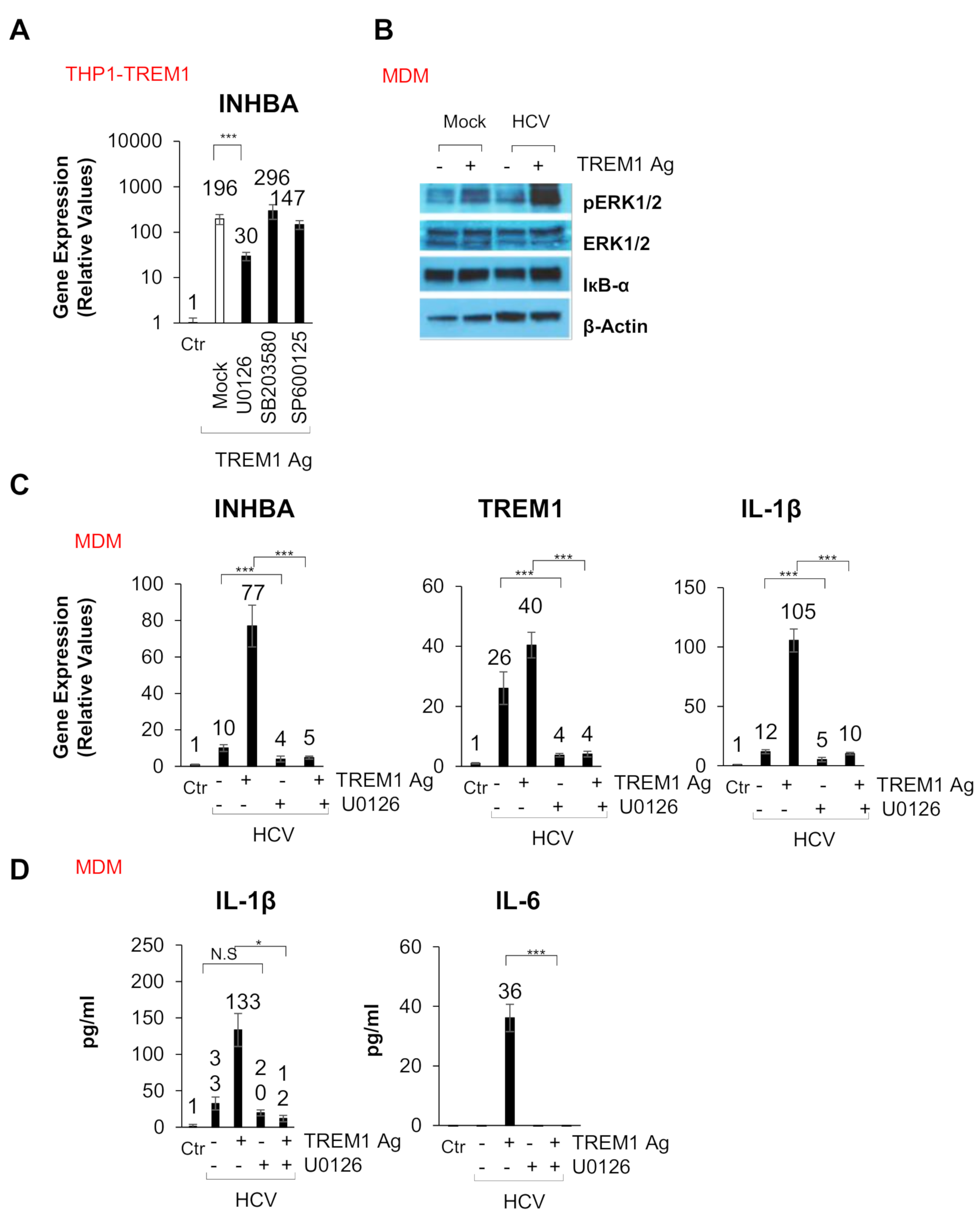

Supplement: S8 Fig — (A) THP1-TREM1 overexpressing cells were treated with the TREM1 agonist (1 μg/mL) ± mock, U0126 (ERK inhibitor, 10 μM), SB203580 (p38 MAPK inhibitor, 10 μM), or SP600125 (JNK inhibitor, 20 μM). Gene expression of INHBA was analyzed by qPCR. (B) MDMs were stimulated with either mock or HCV ± TREM1Ag and protein expression levels of phospho-ERK, total ERK, and IκB-α were analyzed by Western blot. β-actin was used as an internal control. (C) MDMs were stimulated with HCV ± TREM1Ag ± U0126 and gene expression of INHBA, TREM1, and IL-1β were analyzed by qPCR. (D) MDMs were treated as in (C) and IL-1β and IL-6 levels in the supernatant were measured by ELISA. All treatments were performed for 24 hours at a MOI. = 1. For qPCR, results are shown as fold induction compared to control samples after normalizing with 18S internal control. Data from repeated experiments were averaged and are expressed as means ± SD. *P≤0.05, **P≤0.01, ***P≤0.001; ns, non-significant. (TIF) [file ppat.1007883.s008.tif]

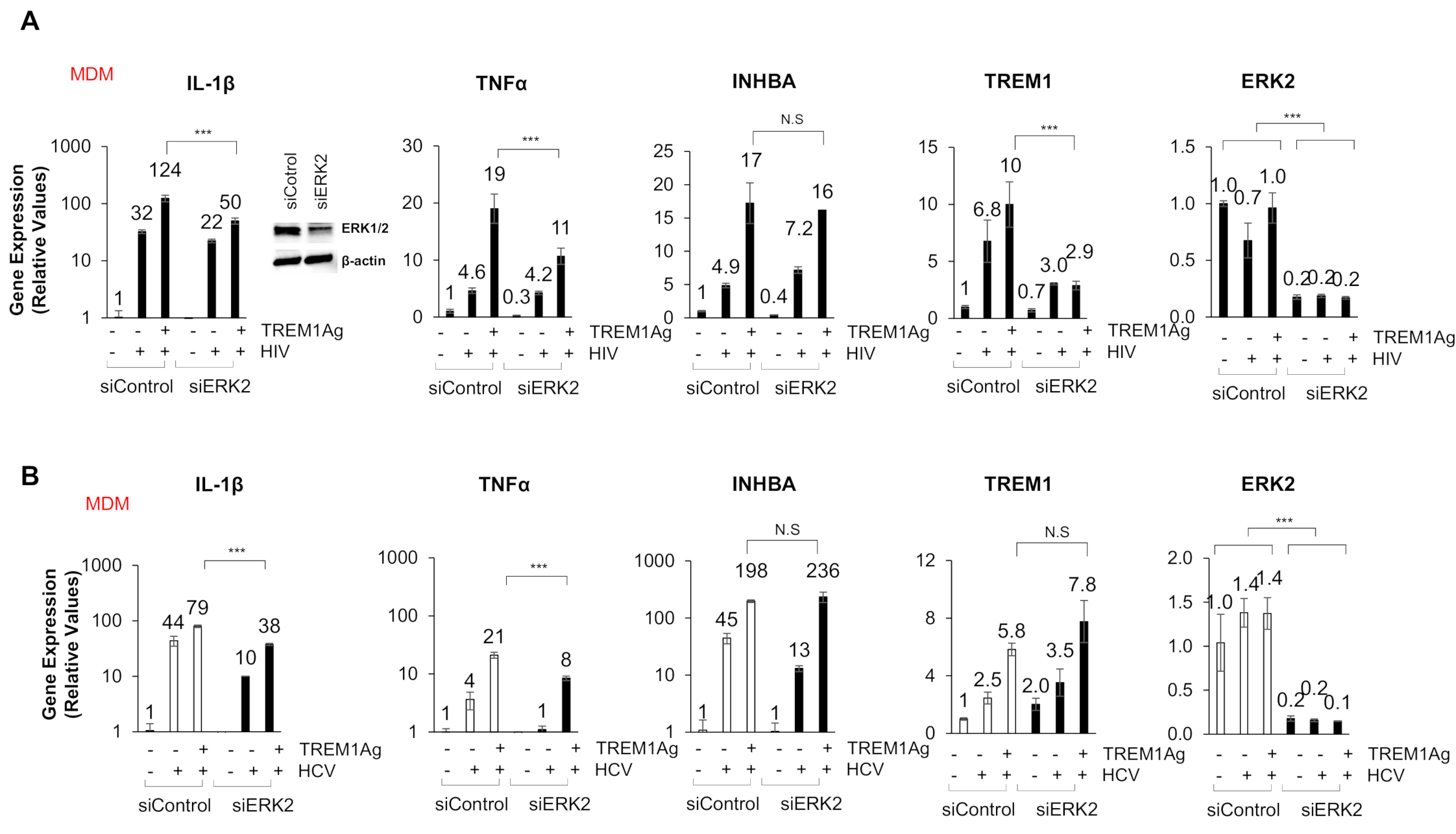

Supplement: S9 Fig — (A) Gene expression of IL-1β, IL-6, TNFα, TREM1, and INHBA in MDMs after silencing with nonspecific control or ERK2 siRNA and stimulation with HIV in the absence or presence of the TREM1 agonist (1ug/ml). Western blot analysis of indicated proteins from MDMs transfected (t) with non-specific (siCtr) or ERK2 siRNA (siERK2). (B) Gene expression of IL-1β, IL-6, TNFα, TREM1, and INHBA in MDMs after silencing with nonspecific control or ERK2 siRNA and stimulation with HCV in the absence or presence of the TREM1 agonist (1ug/ml). For qPCR, results are shown as fold induction compared to control samples after normalizing with 18S internal control. Data from repeated experiments were averaged and are expressed as means ± SD. *P≤0.05, **P≤0.01, ***P≤0.001; ns, non-significant. (TIF) [file ppat.1007883.s009.tif]

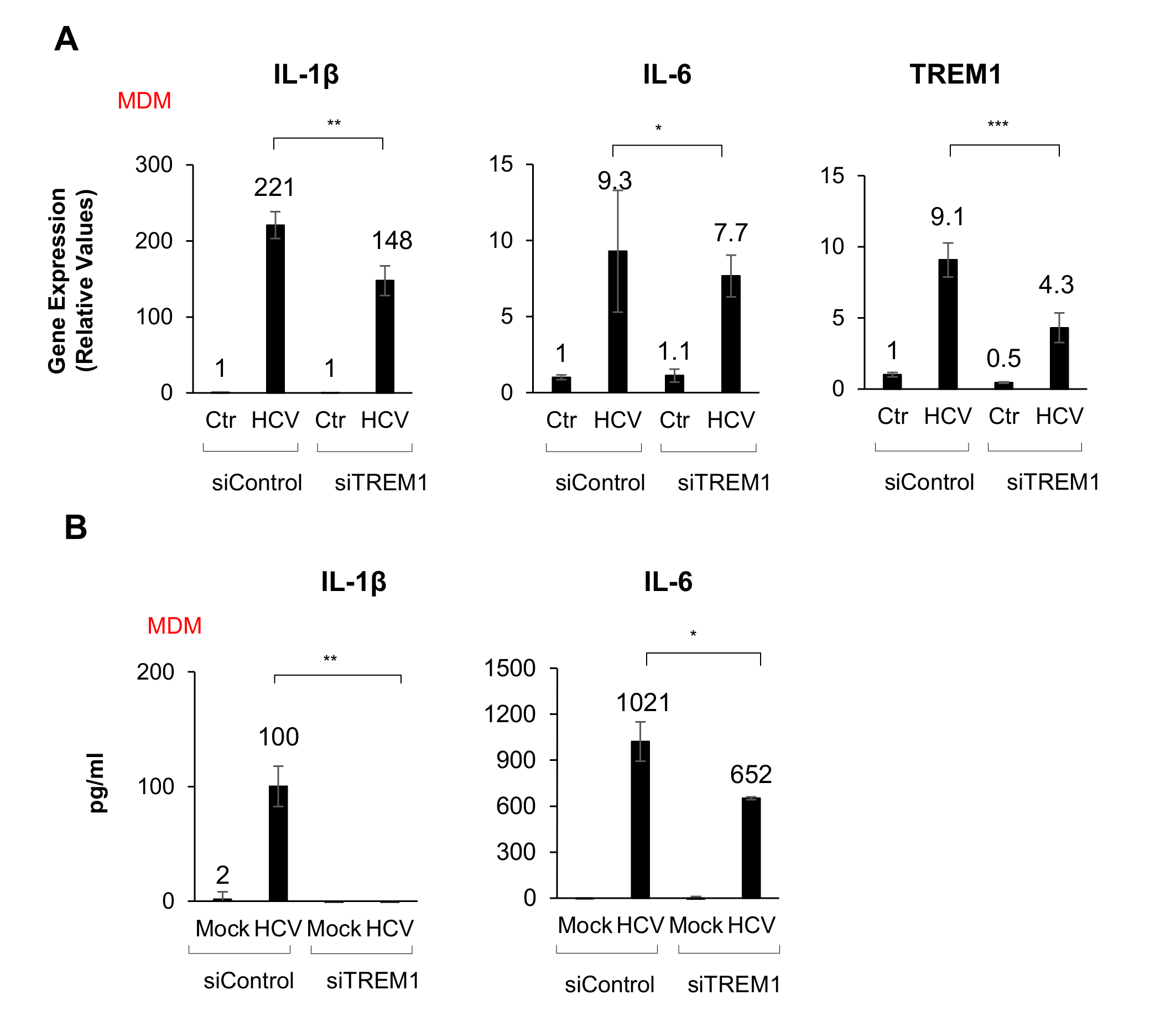

Supplement: S10 Fig — (A) MDMs were treated with control or TREM1 siRNA for 48 hours. Cells were then stimulated with HCV for 24 hours and gene expression of IL-1β, IL-6, and TREM1 were analyzed by qPCR. (B) MDMs were treated as described in (A) and supernatants were analyzed for secretion of IL-1β and IL-6 by ELISA assay. For qPCR, results are shown as fold induction compared to control samples after normalizing with 18S internal control. Data from repeated experiments were averaged and are expressed as means ± SD. *P≤0.05, **P≤0.01, ***P≤0.001; ns, non-significant. (TIF) [file ppat.1007883.s010.tif]

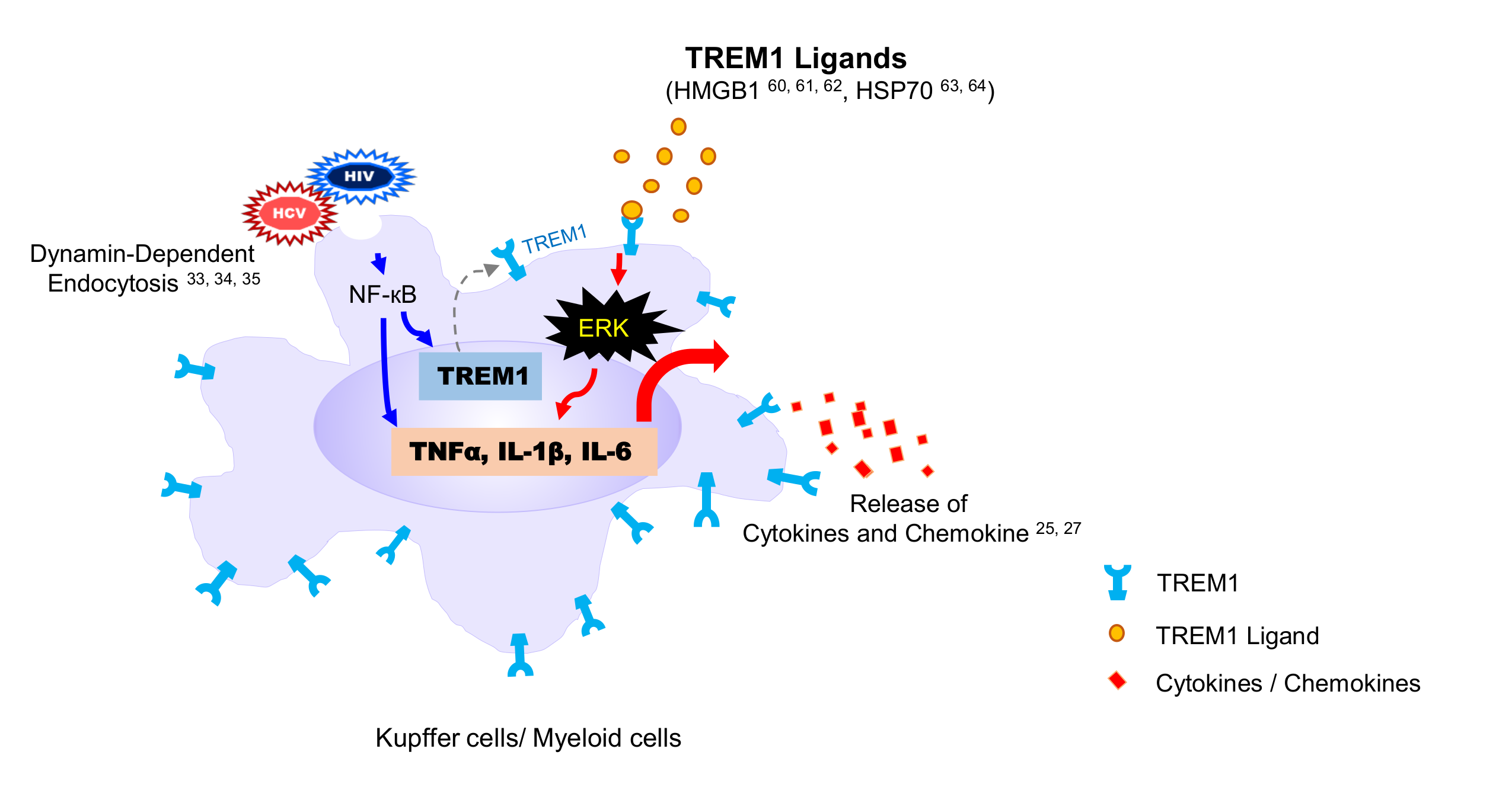

Supplement: S11 Fig — HIV and HCV or viral proteins are internalized by Kupffer or myeloid cells, through dynamin dependent endocytosis, and stimulate upregulation of proinflammatory cytokines and chemokines. These viruses drive upregulation TREM1 expression on the cell surface through NF-кB signaling. Engagement of the TREM1 receptor with its putative ligands, including HMGB1 and HSP70, further activate the ERK1/2 signaling pathway leading to subsequent inflammatory cytokine production. (TIF) [file ppat.1007883.s011.tif]
